# **Evaluation of [**^**13**^**N]ammonia positron emission tomography as a potential method for quantifying glutamine synthetase activity in the human brain**

**DOI:** 10.1186/s13550-020-00731-0

**Published:** 2020-12-03

**Authors:** Alice Egerton, Joel T. Dunn, Nisha Singh, Zilin Yu, Jim O’Doherty, Ivan Koychev, Jessica Webb, Simon Claridge, Federico E. Turkheimer, Paul K. Marsden, Alexander Hammers, Antony Gee

**Affiliations:** 1 Department of Psychosis Studies, Institute of Psychiatry, Psychology and Neuroscience, King’s College London, 16 De Crespigny Park, London, SE5 8AF UK; 2 School of Biomedical Engineering and Imaging Sciences, St Thomas’ Hospital, King’s College London, London, SE1 7EH UK; 3 King’s College London & Guy’s and St. Thomas’ PET Centre, London, SE1 7EH UK; 4 Department of Neuroimaging, Institute of Psychiatry, Psychology and Neuroscience, King’s College London, London, SE5 7AF UK; 5 Present Address: Department of Psychiatry, Warneford Hospital, University of Oxford, Oxford, OX3 7JX UK; 6 Present Address: Clinical Imaging Research Centre, National University of Singapore, Singapore, 117599 Singapore

**Keywords:** Glutamate, Glutamate turnover, PET, Test–retest, Arterial input function, Metabolites, CBF, Cerebral blood flow, One-tissue compartment model

## Abstract

**Purpose:**

The conversion of synaptic glutamate to glutamine in astrocytes by glutamine synthetase (GS) is critical to maintaining healthy brain activity and may be disrupted in several brain disorders. As the GS catalysed conversion of glutamate to glutamine requires ammonia, we evaluated whether [^13^N]ammonia positron emission tomography (PET) could reliability quantify GS activity in humans.

**Methods:**

In this test–retest study, eight healthy volunteers each received two dynamic [^13^N]ammonia PET scans on the morning and afternoon of the same day. Each [^13^N]ammonia scan was preceded by a [^15^O]water PET scan to account for effects of cerebral blood flow (CBF).

**Results:**

Concentrations of radioactive metabolites in arterial blood were available for both sessions in five of the eight subjects. Our results demonstrated that kinetic modelling was unable to reliably distinguish estimates of the kinetic rate constant *k*_3_ (related to GS activity) from *K*_1_ (related to [^13^N]ammonia brain uptake), and indicated a non-negligible back-flux of [^13^N] to blood (*k*_2_). Model selection favoured a reversible one-tissue compartmental model, and [^13^N]ammonia *K*_1_ correlated reliably (*r*^2^ = 0.72–0.92) with [^15^O]water CBF.

**Conclusion:**

The [^13^N]ammonia PET method was unable to reliably estimate GS activity in the human brain but may provide an alternative index of CBF.

## Introduction

The metabolism of glutamate to glutamine by the enzyme glutamine synthetase (GS) is a key process for maintaining healthy synaptic function. GS (encoded by the gene glutamate-ammonia ligase, Glul) is predominantly expressed in astrocytes [1] and converts glutamate released into the synapse during neurotransmission to glutamine, for recycling to neuronal glutamate and gamma-amino butyric acid (GABA). GS is therefore critical to the homeostasis of excitatory and inhibitory neurotransmission and normal brain activity [2, 3]. This process may be compromised in several brain disorders [3], and neuroimaging techniques to assess GS activity in vivo could have wide-ranging research or clinical impact.

Abnormalities in GS have been most clearly linked to epileptogenesis [4]. Very rare inherited deficits in GS are associated with neonatal seizures [5, 6]. Pharmacological inhibition of GS [2] or genetic GS deficiency [7] can be used as animal models of epilepsy, and there are marked reductions in GS in areas of hippocampal tissue resected from patients with mesial temporal lobe epilepsy [8, 9]. Furthermore, regional differences in the level of GS protein, mRNA expression or activity have been detected in post-mortem brain tissue across many psychiatric and neurological disorders. The results of these studies, summarised in Additional file 1: Table S1, suggest that in addition to applications in epilepsy, GS imaging could be important in understanding or predicting schizophrenia, depression or suicidal behaviour, amongst other disorders.

GS is also the main pathway for metabolism of brain ammonia, which is required for the conversion of glutamate of glutamine [10]. This raises the possibility that radiolabelled ammonia in combination with positron emission tomography (PET) may be utilised to measure brain GS activity. [^13^N]Ammonia PET is used clinically to assess myocardial perfusion (“blood flow”) and has been applied in research studies examining abnormalities in brain ammonia uptake associated with liver disease [11–20] and in the diagnosis of brain tumours [21].

The aim of this study was to evaluate [^13^N]ammonia as a PET tracer for quantification of brain GS activity. This evaluation requires kinetic modelling of the dynamic concentrations of ^13^N-derived radioactivity in the brain and arterial blood following radiotracer injection, in an attempt to reliably extract rate constants, as the rate of conversion of [^13^N]ammonia to [^13^N]glutamine, from the signal relating to [^13^N]ammonia brain uptake and clearance (see Additional file 1: Figure S1). To do this we sought to acquire two [^13^N]ammonia scans (test and re-test) in eight healthy volunteers. In order to account for effects of cerebral blood flow (CBF), we additionally acquired test and re-test [^15^O]water PET scans in the same subjects on the same day, with [^15^O]water PET scans preceding [^13^N]ammonia PET.

Primary analysis of the suitability of the method assessed the identifiability and repeatability of the *k*_3_ rate-constant representing GS activity in the kinetic model (see Additional file 1: Figure S1). While this is not sufficient to prove evaluation of GS activity, it would be a pre-requisite to using this standard kinetic modelling approach. Secondary analysis assessed identifiability and repeatability of other parameters, kinetic model selection, and comparisons to previous work using [^13^N]ammonia to evaluate blood–brain-barrier function [11].

## Methods

The study had ethical approval from the NHS Ethical Committee (NRES South East Coast, Surrey), the local Research and Development offices and the Administration of Radioactive Substances Advisory Committee (ARSAC). Participation required provision of written informed consent to all study procedures.

### Participants

The study aimed to acquire complete datasets (including one T1-weighted MRI scan, two [^13^N]ammonia scans and two [^15^O]water PET scans) in eight healthy volunteers. Participants were recruited internally though King’s College London’s recruitment system. Inclusion required that participants were aged 18 or older and were able to provide written informed consent in English. Exclusion criteria included the standard contraindications to PET and MRI, including pregnancy. Absence of pregnancy in female participants was confirmed by a negative urine pregnancy test on arrival to the PET scanning visit.

### MRI

MRI scans were performed at the Centre for Neuroimaging Sciences, King’s College London, UK on a General Electric MR750 3T MRI scanner. A T1-weighted structural MRI scan based on the ADNI protocol (voxel size 1.05 × 1.05 × 1.20 mm, TE 3.016 ms; TR 7.312 ms matrix 256 × 256; FoV 270 mm; inversion time 400 ms) was acquired for co-registration of the participants’ PET images.

### Radiochemistry

Aqueous [^13^N]ammonia was produced on a CTI RDS 112 biomedical cyclotron via the ^16^O(p,α)^13^N nuclear reaction. The target contained 8 mL H_2_O with 5 mM ethanol according to Wieland et al. [22].

[^15^O]water: Oxygen-15 was produced in the form of [^15^O]oxygen gas by the bombardment of enriched [^15^N]nitrogen gas containing 1–2.5% oxygen gas via the ^15^ N(p,n)^16^0 nuclear reaction. [^15^O]water was subsequently obtained by passage with hydrogen over a platinum catalyst according to Berridge et al. [23].

### PET image acquisition

PET scans were acquired at St Thomas’ Hospital, King’s College London on a GE Discovery 710 PET-CT scanner with 3D acquisition and list mode. Each participant underwent two PET scanning sessions, performed in the morning and afternoon of the same day. Each of the two scanning sessions consisted of an initial low dose CT scan to enable correction for tissue attenuation of radioactivity, a dynamic [^15^O]water scan (5 min), and a dynamic [^13^N]ammonia scan (30 min). There was a break of approximately one hour between the two sessions, during which lunch was provided, and an appropriate gap (at least 5 half-lives) between subsequent scans to avoid residual counts (i.e. at least 10 min following the [^15^O]water scans and 50 min following the [^13^N]ammonia scan).

At the start of the PET scan visit, a cannula was inserted in a vein in the arm for radiotracer injection. After application of local anaesthetic, an arterial line was inserted into the radial artery and flushed every 20 min with heparinised saline (20 IU/mL of heparin in sterile 0.9% *w/v* sodium chloride) until removal at the end of PET scanning. Just before the start of each scanning session, 6 mL of arterial blood was taken to measure baseline blood ammonia levels.

Participants were positioned in the PET-CT scanner, with head movement minimised via a moulded headrest and head strap. The arterial cannula was connected to an automated blood sampling system (Allogg ABSS, www.allogg.se, Sweden) using a 150 cm PTFE coated tubing (inner diameter 1 mm). CT scout (0.015 mSv) and CT attenuation correction (0.05 mSv) scans were acquired. ^15^O-water (target dose at time of administration: 960 MBq, 1.10 mSv) was injected through the venous cannula over 10 s. PET image acquisition started 10 s before the start of [^15^O]water injection and continued for a total of 5 min. Arterial blood collection via the fluid analyser commenced 70 s before [^15^O]water injection and 60 s before the start of scan acquisition and continued for the 5 min scan duration, to a total of 25 mL. Additionally, a single 2 mL arterial blood sample was manually drawn at 4 min into the scan.

After completion of the [^15^O]water scan the arterial line was flushed with heparinised saline. At least 20 min after the end of the ^15^O-water scan (25 min after [^15^O]water injection), [^13^N]ammonia (target dose at time of administration: 550 MBq, 1.5 mSv) was injected through the venous cannula. PET image acquisition started 10 s before the start of [^13^N]ammonia injection and continued for 30 min. Arterial blood collection via the fluid analyser commenced 70 s before [^13^N]ammonia injection and 60 s before the start of scan acquisition and continued for 15 min, to a total of 75 mL. In addition, 6 manual arterial blood samples of 10 mL each were drawn at 4, 6, 8, 12, 20 and 30 min after scan start during the [^13^N]ammonia scans, which were used for whole blood, plasma and metabolite analysis.

In the second session, a minimum of 1 h later, both the [^15^O]water and [^13^N]ammonia scans were repeated using identical acquisition protocols.

### Ammonia and metabolite analysis

The method used for separation of ammonia and metabolite from plasma samples was based on the method published by Keiding et al. [11] and is described below.

Levels of non-radioactive ammonia in arterial blood were determined from samples collected before radiotracer collection. These samples were collected in K-EDTA tubes (pre-tested and confirmed as ammonia-free) and transported on ice within 20 min of collection to the hospital laboratory for standard analysis.

Unless stated otherwise, all water used in these metabolite analyses was passed through ion exchange resin and 0.22 μm membrane filtered to produce water with a specific resistance of 18.2 micro-ohms using a Milli-Q Ultrapure water purification system manufactured by Millipore Corporation.

Plasma was separated from whole blood by centrifuging at 3000 × *g* for 3 min at room temperature (RT). Levels of radioactive metabolites in plasma were estimated through solid phase extraction, based on the methods of Keiding et al. [17]. In preparation for solid phase extraction, one cartridge was filled with 0.6 mL Dowex 1X8-50 anion exchange resin and pre-treated with 6 mL 0.75 M sodium acetate solution. A second cartridge, connected in series via an Agilent Bond Elut adapter, was filled with 0.35 mL AG50W-X8 cation exchange resin and pretreated with 3.5 mL 0.8 M Tris–acetate solution. The third cartridge which connected to the second cartridge in the same way via adapter was filled with 0.35 mL AG50W-X8 cation exchange resin and pretreated with 3.5 mL Millipore water.

For extraction, 0.5 mL of the supernatant protein-free plasma was loaded onto the first cartridge followed by washing with 3 mL of Millipore water through the cartridge stack and flushed with 10 mL of air. The eluent from the first cartridge passed through the second cartridge and third cartridge, which were subsequently washed with 7 mL of Millipore water followed by 10 mL of air. The third cartridge was washed with 7 ml Millipore water and followed by 10 mL of air. All eluates were collected with a 25 mL pot. With this method, the radioactivity measured on the first cartridge corresponded to [^13^N]glutamate, on the second cartridge corresponded to intact [^13^N]ammonia, on the third cartridge corresponded to [^13^N]glutamine, and the pot corresponded to [^13^N]urea.

A 10-detector gamma-counter (Wizard2 2470, Perkin-Elmer) cross-calibrated to the PET scanner was used to measure radioactivity concentrations in whole blood (0.5 mL per sample), plasma (0.5 mL per sample) and metabolite fractions (3 mL for urea and full cartridge contents for other fractions). All samples were counted for 3 min on a fixed energy window (358–664 keV) with software cross-talk correction and in-house volumetric geometry correction. The samples and cartridges were corrected for weight to calculate the total radioactivity of blood sample analysed. All sample data were background and decay corrected to scan start time prior to data analysis.

### Image processing

[^15^O]water PET list mode data was unlisted to 26 frames (1 × 10 s, 10 × s, 6 × 10 s and 9 × 20 s). [^13^N]ammonia PET list mode was unlisted to 47 frames (1 × 10 s, 10 × 5 s, 6 × 10 s, 3 × 20 s, 27 × 60 s). All PET images were reconstructed to a 256^2^ matrix with 47 slices with 0.98 × 0.98 × 3.27 mm voxel size, 3D iterative reconstruction (GE “VuePoint”, 4 iterations, 24 subsets, 4 mm FHWM Gaussian post-filter), scatter correction and inter- and intra-frame decay correction. Images were reconstructed with CT attenuation correction (attenuation corrected, AC) and without (non-attenuation corrected, NAC).

Frame-by-frame motion correction was performed on dynamic PET data using the NAC image to derive the rigid-body motion parameters which were applied to the paired AC image (first 9 frames ignored to avoid low counts). Regions of interest (ROI) were defined by the “Hammers_mith Atlas” [24, 25] (83 regions) in MNI stereotaxic space. Nonlinear warps from MNI to subject space were defined using the unified segmentation algorithm [26] in SPM8 (www.fil.ucl.ac.uk/spm) on each subject’s T1 MRI. Resliced atlases for each subject were then co-registered to a summed PET image (sum of total scan duration of motion corrected AC image ignoring first 60 s) for each PET scan via the MRI.

For both the [^15^O]water and [^13^N]ammonia scans, time activity curves (TACs) were extracted from the co-registered Hammers_mith atlas [24, 25] (ignoring ventricular and white matter regions). Using each subject’s co-registered probabilistic grey matter mask from the segmented MRI, TACs were extracted using the mean voxel value within the region or a weighted mean for cortical regions using each subject’s grey matter probabilistic mask. Whole-brain grey matter and white matter weighted mean TACs were also defined. A total of 79 regions were explored (77 atlas ROIs plus global grey and white matter).

### Blood data processing

For the [^13^N]ammonia scans, arterial whole blood input functions were created from decay-corrected continuous blood samples with manual samples used for cross-calibration to scanner and interpolation to scan end. Plasma-over-blood ratio was calculated as the mean of the manual plasma and whole blood sample ratios for each subject (Additional file 1: Figure S2). Parent fraction data (ratio of [^13^N]ammonia to total ^13^N activity) was fitted to a biexponential curve for each subject as used by Keiding et al. [11]. Parent plasma input functions (i.e. [^13^N]ammonia in plasma only) for the kinetic modelling were created by multiplying the whole-blood input function by plasma-over-blood ratios and the biexponential curve fitted to the parent fractions. To account for delay between the blood sampling detector and PET scan whole blood and parent plasma input functions were delay corrected by visually matching the blood rise with the grey matter TAC, with decay correction.

### Kinetic analysis

Regional cerebral blood flow (CBF) was calculated from the [^15^O]water TACs using the 5-parameter free diffusion model as described by Meyer [27] applied to each time activity curve. In brief, a nonlinear least squares fit method was used to simultaneously estimate the 5 free parameters of this 1-tissue compartment model: CBF, $k\prime_{2}$ (^15^O wash-out), blood fraction, and delay and dispersion of the blood curve between the brain and sampling point (in addition to the visual delay correction described above).

Ammonia is a freely diffusible tracer and as such has been used to quantify perfusion in myocardium [28] and brain [29]. Though ammonia is rapidly trapped in tissue, in order to index GS activity, the kinetic parameters describing the uptake of [^13^N]ammonia by GS must be distinguishable from those reflecting CBF. The model chosen for primary analysis of [^13^N]ammonia scans was an irreversible two tissue compartment model (2TCM) as used in Keiding et al. [11]. To confirm the model choice a nonlinear spectral analysis approach was used to identify the most appropriate tissue uptake model [30]. In brief, the data was fitted to a number of candidate PET compartmental models with increasing numbers of parameters. In this case, a reversible 2TCM [Additional file 1: Figure S1] was the most complex model considered, with increasingly simpler models defined by setting *k*_4_, *k*_3_, *k*_2_ to zero (i.e. 4 candidate models). The blood fraction contributing to the TAC for each region was also included as a free parameter.

Each compartmental model was fitted using a weighted least squares method with weighting inversely proportional to the variance of each frame determined by frame duration and radioactive decay: ${{\Delta }_{i}e}^{-\lambda {t}_{i}}$, where $\lambda$ is the decay rate constant, and ${\Delta }_{i}$ and ${t}_{i}$ are the frame duration and frame mid-point time, respectively, for frame $i$.

Additional macroparameters from the ^15^O and ^13^N scans were calculated to compare with the results of Keiding et al. [11]. These parameters are not directly of interest to the identification of the *k*_3_ parameter, but were obtained solely for the purpose of comparison. PS_BBB_ (flow independent permeability-surface area product of the blood brain barrier to [^13^N]ammonia) was calculated as

$${\text{PS}}_{{{\text{BBB}}}} = - {\text{CBF}} \ln \left( {1 - K_{1} /{\text{CBF}}} \right)$$

where CBF is calculated from the [^15^O]water scan (assuming a 100% extraction fraction), and *K*_1_ from the [^13^N]ammonia scan. Extraction Fraction (EF) was also calculated as the simple ratio of *K*_1_ to CBF. Net metabolic clearance of [^13^N]ammonia in blood into intracellular [^13^N]glutamine, *K*_met_, was calculated using the Patlak graphical method using the complete data, with a t* of 20 min [32]. PS_met_ (flow-independent permeability-surface area product of conversion of ammonia to intracellular glutamine) was calculated as

$${\text{PS}}_{met} = - {\text{CBF}} \ln \left( {1 - K_{{{\text{met}}}} /{\text{CBF}}} \right)$$

Finally, metabolic flux of ammonia molecules from blood to glutamine in tissue, Flux_met_, (as described by Keiding et al. [11]), was calculated as

$${\text{Flux}}_{{{\text{met}}}} = K_{{{\text{met}}}} A$$

where *A* is the measured concentration of endogenous ammonia in the blood.

### Statistical analysis

Identifiability of the *k*_3_ rate-constant from the [^13^N]ammonia data was evaluated by testing if the estimated parameter value, relative to estimated error, was significantly greater than zero (using the one-sided *t*-test). For the irreversible 2TCM model (4 parameters) with 47 frames, this corresponds to a proportional estimate parameter error of 39%. In addition, optimal model selection on the [^13^N]ammonia data was assessed using the Akaike Information Criterion (AIC) [31].

Kinetic parameter repeatability between the test–retest scans was assessed using mean fractional difference (VAR), absolute fractional difference (AbsVAR), and intraclass correlation coefficient (ICC) using a two-way random model for consistency [33]. For 8 subjects, the threshold for a significantly positive ICC is 0.58 at the *p* < 0.05 level. VAR and AbsVAR were calculated for N subjects as a percentage:

$$\begin{aligned} & {\text{VAR}} = \frac{1}{N} \mathop \sum \limits_{i = 1}^{N} 200 \times \frac{{{\text{retestValue}}_{i} - {\text{testValue}}_{i} }}{{{\text{testValue}}_{i} + {\text{retestValue}}_{i} }} \\ & {\text{AbsVAR}} = \frac{1}{N} \mathop \sum \limits_{i = 1}^{N} 200 \times \frac{{\left| { {\text{testValue}}_{i} - {\text{retestValue}}_{i} } \right|}}{{{\text{testValue}}_{i} + {\text{retestValue}}_{i} }} \\ \end{aligned}$$

Image registration, TAC extraction, blood data processing, kinetic modeling and statistical analyses were performed in MATLAB (www.mathworks.com). Data are presented as mean ± s.d. unless otherwise stated.

## Results

### Participants

Eight volunteers (3 female) underwent all PET-CT and MRI scans. Age at scan was 25.0 ± 2.5 years. Weight, height and BMI were 75.7 ± 9.3 kg, 1.74 ± 0.13 m and 25.3 ± 4.1 kg/m^2^, respectively.

### Scan parameters

Ammonia blood levels immediately prior to scan session one were 24.5 ± 5.7 µmol/L and prior to session two 23.9 ± 4.8 µmol/L. No significant difference was found between scan session within-subject (0.6 ± 7.9 µmol/L; range − 12.0 to 11.0). No significant differences were observed for the administered [^15^O]water (mean ± s.d. = 841 ± 126 MBq, and 834 ± 146 MBq for sessions one and two, respectively) or the [^13^N]ammonia (mean ± s.d. = 537 ± 7 MBq and 537 ± 3 MBq for sessions one and two, respectively). Additionally, no significant differences were observed between the timings between sessions one and two. The mean time between [^15^O]water and [^13^N]ammonia injections was 48 ± 6 min and 46 ± 4 min for sessions one and two, respectively, with a minimum gap of 37 min. The mean time between first [^13^N]ammonia and second [^15^O]water injections was 88 ± 33 min (minimum 65 min).

### [^15^O]water: kinetic analysis and repeatability

Visual inspection indicated that grey matter TAC fits were excellent (Additional file 1: Figure S2). Grey matter CBF was 37.6 ± 4.8 and 38.5 ± 3.4 mL/100 g/min for sessions one and two, respectively (Table 1, no significant difference). Repeatability of CBF calculations across 8 subjects was moderate (Table 2) and ICC values were significantly > 0 in 44 of 77 individual grey matter ROIs (mean ICC = 0.58 ± 0.23) and the white matter, but not in the whole brain grey matter TACs (ICC = 0.50). VAR and AbsVAR were 2.7% and 8.6%, respectively, for grey matter. Across individual ROIs, the mean ± s.d. values for VAR and AbsVAR were 2.3 ± 2.5% and 9.7 ± 2.5%, with values below 10% in 50 of 77 ROIs. Statistics for all parameters in this model are shown in Additional file 1: Tables S4 and S5 with TACs and fits in Additional file 1: Figure S6.

**Table 1:** Model parameters for test (S1) and retest (S2) scans for grey matter and selected regions of interest (ROIs)

ROI	[^15^O]water [*n* = 8]		[^13^N]ammonia [*n* = 5]					
	CBF (mL/100 g/min)		*K*_1_ (mL/100 g/min)		*k*_2_ (1/min)		*k*_3_ (1/min)	
	S1	S2	S1	S2	S1	S2	S1	S2
Grey Matter	37.6 ± 4.8	38.5 ± 3.4	20.1 ± 1.5	18.7 ± 1.3	0.0072 ± 0.0035	0.0096 ± 0.0042	0.0031 ± 0.0045	0.0177 ± 0.0259
White Matter	25.8 ± 3.4	26.4 ± 2.7	14.6 ± 1.1	13.7 ± 0.7	0.0073 ± 0.0043	0.0096 ± 0.0053	0.0139 ± 0.0177	0.0274 ± 0.0401
Hippocampus_l	32.1 ± 3.7	33.8 ± 3.1	19.7 ± 2.8	18.8 ± 1.6	0.0256 ± 0.0145	0.0360 ± 0.0212	0.0532 ± 0.0261	0.0621 ± 0.0304
OL_ling_G_l	40.8 ± 5.4	41.5 ± 2.8	23.7 ± 3.0	21.6 ± 0.9	0.0072 ± 0.0023	0.0090 ± 0.0035	0.0000 ± 0.0000	0.0125 ± 0.0172
G_cing_post_l	43.6 ± 5.3	44.3 ± 4.2	22.2 ± 1.6	20.8 ± 1.3	0.0069 ± 0.0038	0.0099 ± 0.0059	0.0038 ± 0.0052	0.0154 ± 0.0231
Putamen_l	45.3 ± 7.3	46.1 ± 5.5	23.3 ± 1.9	21.2 ± 1.5	0.0063 ± 0.0028	0.0086 ± 0.0037	0.0000 ± 0.0000	0.0144 ± 0.0203
Thalamus_l	43.9 ± 5.5	44.5 ± 4.2	22.8 ± 2.4	21.2 ± 1.2	0.0128 ± 0.0057	0.0178 ± 0.0075	0.0241 ± 0.0214	0.0405 ± 0.0234
Cerebellum_l	41.7 ± 6.8	42.6 ± 5.2	22.5 ± 2.0	20.7 ± 1.3	0.0070 ± 0.0047	0.0088 ± 0.0034	0.0056 ± 0.0126	0.0145 ± 0.0216

The table presents estimates of cerebral blood flow (CBF) from [^15^O]water scans, and irreversible two tissue compartmental model parameter estimates for rate constants *K*_1_, *k*_2_ and *k*_3_ from [^13^N]ammonia scans. Data are presented as mean ± standard deviation for grey matter, white matter and the left (l) hippocampus, lingual gyrus (OL_ling_G), posterior cingulate gyrus (G_cing_post), putamen, thalamus, and cerebellum

**Table 2:** Repeatability metrics for [^15^O]water cerebral blood flow (CBF) and [^13^N]ammonia two tissue compartment model parameters for grey matter and selected regions of interest (ROIs)

	[^15^O]water [*n* = 8]			[^13^N]ammonia [*n* = 5]								
ROI	CBF			*K*_1_			*k*_2_			*k*_3_		
	ICC	VAR	AbsVAR	ICC	VAR	AbsVAR	ICC	VAR	AbsVAR	ICC	VAR	AbsVAR
Grey Matter	0.50	3 ± 11	9 ± 6	− 0.79	− 7 ± 13	10 ± 11	0.94	29 ± 18	29 ± 18	0.02	− 30 ± 179	153 ± 62
White Matter	0.69	2 ± 10	7 ± 6	− 0.89	− 6 ± 13	9 ± 10	0.93	28 ± 18	28 ± 18	− 0.05	− 46 ± 163	141 ± 67
Hippocampus_l	0.17	5 ± 13	11 ± 9	0.35	− 4 ± 13	10 ± 8	0.62	34 ± 44	48 ± 23	0.22	15 ± 49	39 ± 27
OL_ling_G_l	0.22	2 ± 13	10 ± 9	− 0.42	− 8 ± 15	10 ± 14	0.86	21 ± 13	21 ± 13	− 0.00	71 ± 165	147 ± 80
G_cing_post_l	0.68	2 ± 9	8 ± 4	− 0.78	− 7 ± 13	9 ± 11	0.86	34 ± 24	34 ± 24	0.15	13 ± 196	173 ± 37
Putamen_l	0.85	2 ± 8	7 ± 2	− 0.84	− 9 ± 14	12 ± 12	0.85	31 ± 24	31 ± 24	− 0.00	59 ± 194	181 ± 33
Thalamus_l	0.66	2 ± 9	7 ± 5	− 0.64	− 7 ± 15	11 ± 11	0.76	35 ± 27	37 ± 25	0.42	80 ± 95	105 ± 57
Cerebellum_l	0.69	3 ± 12	9 ± 7	− 0.58	− 8 ± 13	9 ± 13	0.91	30 ± 27	32 ± 24	0.22	− 58 ± 157	138 ± 73

The table presents the intraclass correlation coefficient (ICC), percent mean fractional difference (VAR) and percent absolute fractional difference (AbsVAR) across test and retest scans. Data are presented as mean ± standard deviation for grey matter, white matter and the left (l) hippocampus, lingual gyrus (OL_ling_G), posterior cingulate gyrus (G_cing_post), putamen, thalamus, and cerebellum

### [^13^N]ammonia: metabolite analysis

**Fig. 1:**
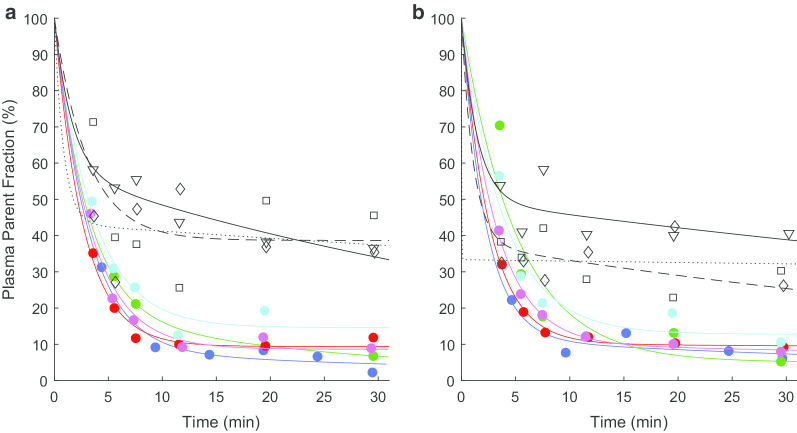
Plasma parent fraction data and fit. Plasma parent fraction for **a** test scan and **b** retest scan for each subject. Symbols represent ratio of parent compound, [^13^N]ammonia, to total ^13^N labelled fractions (parent plus metabolites) measured from individual arterial plasma samples. Lines show biexponential fits to subject/scan data sets. Coloured lines and circles show colour-coded subjects 1–5 used in the full analysis (blue, green, red, magenta and cyan, for subjects 1–5, respectively). Black symbols and lines (triangle/solid, square/dashed, diamond/dotted for subjects 6–8, respectively) show subjects 6–8 considered corrupted data. *X*-axis is time in minutes from injection (corrected for brain-detector delay). *Y*-axis is plasma parent fraction in percent

Continuous and manual blood sampling was obtained for all scans in all 8 subjects. [^13^N]ammonia and [^13^N]metabolite fractions were successfully obtained for the first five subjects but appeared as outliers in the subsequent three subjects (Fig. 1). The source of these outliers is unknown but potentially due to issues with later preparation of solid phase extraction materials. In the first 5 subjects, biexponential curve fits to parent fractions were in line with results from Keiding et al. [11]. Data from these 5 subjects were used to determine the [^13^N]ammonia kinetic model parameter fits and repeatability. Plasma over blood fractions are shown in Additional file 1: Figure S2. Fits were only performed to the parent fraction though individual ^13^N labelled compound fractions are shown in supplementary data (Additional file 1: Figure S3). For all subjects, input functions with grey matter TACs for comparison are shown in Additional file 1: Figures S7 and S8.

### [^13^N]ammonia: *k*_3_ identifiability and model selection

Across 5 subjects, 2 scans and 79 ROIs, 790 TACs were analysed using the irreversible 2TCM [11]. No region showed a nonzero *k*_3_ value across all subjects and scans, indicating trapping of ^13^N was either inconsistent or negligible. Overall, only 54/790 TACs estimated significantly positive *k*_3_ (*p* < 0.05), but this was not consistent across pairs of scans. For comparison, by the same criteria, *K*_1_ was nonzero in 779/790 regions, and *k*_2_ in 452/790 (Additional file 1: Table S12).

Using AIC to compare the results of alternative candidate kinetic model fits, no regions showed best fits to the irreversible 2TCM consistently across all subjects and scan pairs, again reflecting negligible trapping. Of 790 TACs, 656 fit the reversible 1TCM best, with 109 preferring the standard irreversible 2TCM. Looking at consistency of model choice across scan pairs, 70 of 79 regions selected the reversible 1TCM across a majority of subjects Only one region, left hippocampus, selected the irreversible 2TCM in a majority of subjects: 3/5 (this was also the case when including all 8 subjects: 5/8). Note, in this region, *k*_3_ ICC and interscan variability was also poor (Table 1). The remaining models showed no consistent model preference across subjects and scans: 24/790 TACs preferred irreversible 1TCM and 1/790 preferred reversible 2TCM (Additional file 1: Table S13, with graphical representation across all subjects and regions in Additional file 1: Figure S4).

### [^13^N]ammonia: kinetic analysis and repeatability

**Fig. 2:**
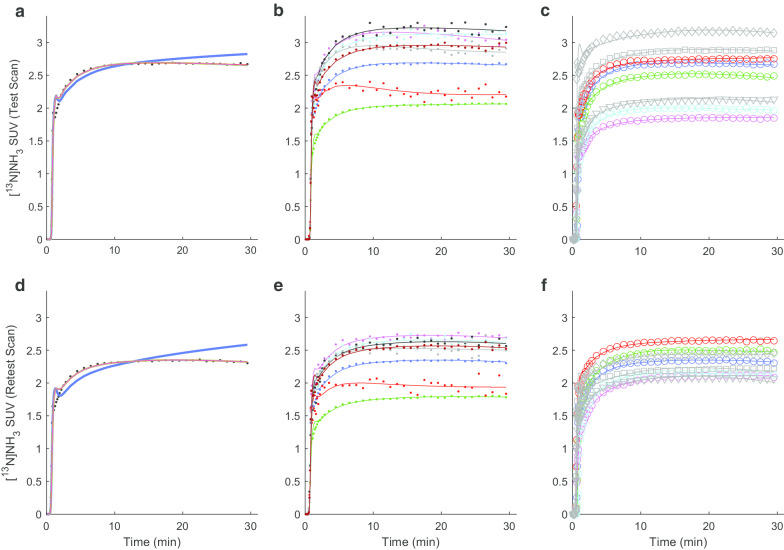
Representative ammonia TAC data and fit examples. Symbols show example time activity curves (TACs) from ammonia PET scans and lines show model fits. Top row (**a**–**c**): data from test scan; bottom row (**d**–**f**) data from retest scan. 1st column (**a**, **d**): Subject 1, fits to grey matter TAC: blue line: irreversible 1TCM (*K*_1_ plus blood volume, *V*_b_); green line: reversible 1TCM (*K*_1_, *k*_2_, *V*_b_); red line: irreversible 2TCM (*K*_1_, *k*_2_, *k*_3_, *V*_b_) and magenta line: reversible 2TCM (*K*_1_, *k*_2_, *k*_3_, *k*_4_, *V*_b_). The most complex three models appear to fit equally well and appear as superimposed on the graph. 2nd column (**b**, **e**): TAC (point) and irreversible 2TCM model fits (lines) from subject 1 for grey matter (blue), white matter (green), left hippocampus (red), left lingual gyrus (magenta), left posterior cingulate gyrus (cyan), left putamen (black), left thalamus (grey) and left cerebellum (dark red). **c** TACs (points) and irreversible 2TCM model fits (lines) for grey matter in all subjects. Coloured circles and lines (blue, green, red, magenta and cyan, for subjects 1–5, respectively) and grey symbols and lines (triangle/solid, square/dashed, diamond/dotted for subjects 6–8, respectively). All fits for all subjects are shown in Additional file 1: Figure S5

[^13^N]ammonia PET images showed regional variation in signal intensity, with highest uptake in the occipital lobes, posterior cingulate gyri, putamina, thalami and cerebella. Figure 2 shows example TACs and fits (further data in Additional file 1: Figure S5). Table 1 presents *K*_1_, *k*_2_ and *k*_3_ values for the [^13^N]ammonia scans from subjects 1–5, calculated using the irreversible 2TCM across grey matter and in selected high uptake ROI in the left hemisphere (additional statistics in Additional file 1: Tables S6–S9). *K*_1_ values were comparable between subjects and scan sessions. *K*_1_ ICC values (Table 2) were negative indicating poor repeatability, though absolute fractional differences were comparable to those of CBF, indicating the negative ICC values are a consequence of low intersubject variability. Values for washout (*k*_2_) and trapping (*k*_3_) rate constants (Table 1) were small and highly variable across region, subject and scan. ICC values were high for *k*_2_ despite large differences between the test and retest scans (Table 2). This is likely to be due to the even larger variation in *k*_2_ values across subjects rather than reflecting strong test–retest repeatability. Test–retest variation in the trapping rate constant *k*_3_ was extremely poor (Table 2).

The results from the majority-preferred reversible 1TCM are shown in Table 3 (further statistics in Additional file 1: Tables S10 and S11). The repeatability metrics for *K*_1_ and *k*_2_ calculated using the 1TCM (Table 3) were similar to those calculated using the 2TCM (Table 2). *K*_1_ continued to show low or negative ICC values and fractional differences of approximately 10%; *k*_2_ showed higher ICC but larger inter-subject variability and test–retest differences. Volume of Distribution (*K*_1_/*k*_2_) was also calculated but this too had high intrasubject variability.

**Table 3:** Repeatability metrics for [^13^N]ammonia one-tissue compartment (reversible) model parameters for grey matter and selected regions of interest (ROIs)

ROI	*K*_1_ [*n* = 5] (mL/100 g/min)					*k*_2_ [*n* = 5] (1/min)				
	S1	S2	ICC	VAR	AbsVAR	S1	S2	ICC	VAR	AbsVAR
Grey Matter	20.1 ± 1.5	18.6 ± 1.3	− 0.83	− 8 ± 14	10 ± 12	0.0069 ± 0.0029	0.0074 ± 0.0022	0.58	11 ± 38	28 ± 24
White matter	14.5 ± 1.2	13.5 ± 0.8	− 0.89	− 7 ± 14	9 ± 11	0.0059 ± 0.0027	0.0064 ± 0.0021	0.57	12 ± 43	32 ± 27
Hippocampus_l	18.9 ± 2.4	17.6 ± 1.0	− 0.06	− 7 ± 14	10 ± 11	0.0122 ± 0.0061	0.0144 ± 0.0041	0.89	23 ± 27	25 ± 24
OL_ling_G_l	23.7 ± 3.0	21.5 ± 1.0	− 0.41	− 9 ± 15	10 ± 14	0.0072 ± 0.0023	0.0075 ± 0.0020	0.58	5 ± 30	24 ± 15
G_cing_post_l	22.2 ± 1.6	20.6 ± 1.3	− 0.68	− 7 ± 13	9 ± 11	0.0065 ± 0.0032	0.0075 ± 0.0019	0.58	19 ± 40	30 ± 31
Putamen_l	23.3 ± 1.9	21.1 ± 1.5	− 0.83	− 10 ± 15	12 ± 12	0.0063 ± 0.0028	0.0072 ± 0.0028	0.59	13 ± 44	34 ± 27
Thalamus_l	22.5 ± 2.1	20.7 ± 1.1	− 0.79	− 8 ± 15	10 ± 13	0.0089 ± 0.0030	0.0101 ± 0.0024	0.72	15 ± 23	24 ± 9
Cerebellum_l	22.4 ± 2.0	20.6 ± 1.3	− 0.69	− 8 ± 14	9 ± 13	0.0061 ± 0.0027	0.0072 ± 0.0022	0.61	19 ± 39	33 ± 25

The table presents the intraclass correlation coefficient (ICC), percent mean fractional difference (VAR) and percent absolute fractional difference (AbsVAR) across test (S1) and retest (S2) scans. Data are presented as mean ± standard deviation for grey matter, white matter and the left (l) hippocampus, lingual gyrus (OL_ling_G), posterior cingulate gyrus (G_cing_post), putamen, thalamus, and cerebellum

Including data from all 8 subjects (Additional file 1: Tables S8 and S9) did not improve repeatability.

The values obtained for *K*_met_, PS_met_ and Flux_met_ showed quite high fractional differences across test and retest scans (Additional file 1: Table S2). PS_bbb_ and EF showed lower intrasubject variability but also low ICC values (Additional file 1: Table S3).

**Fig. 3:**
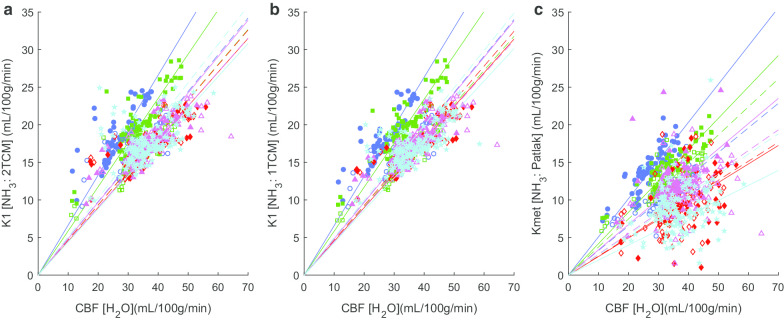
CBF vs *K*_1_ correlations. Plots showing association across 78 ROIs of *K*_1_ (*y*-axis) from model fits of [^13^N]ammonia data with CBF (*x*-axis) from paired [^15^O]H_2_O scans within each subject (1–5) and for scans 1 and 2. Lines show line of best fit (zero intercept). Subjects 1–5 are coded with symbol type and colour. Solid symbols and solid lines show scan 1, open symbols and dashed lines scan 2. **a** *K*_1_ calculated from irreversible 2TCM; **b** *K*_1_ calculated from reversible 1TCM; **c** *K*_met_ calculated from Patlak plot. CBF is calculated using the Meyer [27] model described in the manuscript

Finally, the degree to which CBF may have accounted for brain uptake of [^13^N]ammonia was investigated by examining the correlations between [^15^O]water CBF and [^13^N]ammonia *K*_1_ (as estimated through the irreversible 2TCM or reversible 1TCM) or *K*_met_. Correlating within each scan pair (across all ROIs), *K*_1_ estimates from 1TCM were most highly correlated with CBF in all 10 scan pairs, with *r*^2^ values ranging from 0.72 to 0.92 (Fig. 3). In each of the 10 scan pairs, *r*^2^ values were second highest with the 2TCM *K*_1_ (*r*^2^ between 0.56 and 0.85) and poorest and most variable with *K*_met_ (*r*^2^ between 0.2 and 0.85). However, these strong within-subject correlations were not replicated across subjects within region: no region revealed a significant positive correlation between CBF and *K*_1_ or *K*_met_.

## Discussion

This study evaluated kinetic models for [^13^N]ammonia PET as an in vivo method to assess whether a reliable estimate of trapping could be determined which could be a basis for estimating the rate of conversion of glutamate to glutamine by the enzyme glutamine synthetase (GS) in the human brain. We were able to acquire full datasets comprising two [^13^N]ammonia (test and retest) scans, two [^15^O]water scans and corresponding arterial input functions in five subjects, each on a single day. Kinetic modelling in these subjects was unable to reliably estimate the rate constant relating to GS activity (*k*_3_) from that related to [^13^N]ammonia brain uptake (*K*_1_) and indicated non-negligible back-flux of [^13^N] from the brain to the blood. In addition, comparison of *K*_1_ estimates with [^15^O]water CBF across brain regions and within-subjects found that these measures were highly correlated and of comparable reliability. Together these results indicate that the applied [^13^N]ammonia PET method is unable to quantify GS activity in the human brain, and instead may principally index CBF.

Studies in experimental animals have indicated [^13^N]ammonia PET might be able to index GS activity, as irreversible blockade of GS with methionine sulfoximine (MSO) decreases the brain [^13^N]ammonia signal [34]. In kinetic modelling of dynamic [^13^N]ammonia PET images of the human brain, GS activity would be captured by the rate constant *k*_3_ in an irreversible two tissue compartment model. Using this model, our analysis returned values for *k*_3_ that were highly variable within subjects, as well as between subjects and across grey matter regions. In most instances, *k*_3_ values were also too low to be estimated compared to the estimated error. Only one region of 79, left hippocampus, favoured a model including a *k*_3_ parameter in a majority of subjects, however, considering the small number (3/5) and the poor estimability of *k*_3_ in this region it is possible this is a false positive, though we present the data for future interest. A previous [^13^N]ammonia study in subjects with cirrhosis and healthy volunteers using similar methodology was also unable to provide estimates of *k*_3_ [20]. While volume of distribution (*V*_T_ = *K*_1_/*k*_2_) may potentially have provided a surrogate index of GS activity from a reversible model, we were also unable to reliably estimate *k*_2_, with absolute variability of both *k*_2_ and V_T_ around 30% (Additional file 1: Table S11). Overall, this indicates that [^13^N]ammonia PET is unlikely to be a suitable method for measuring the rate of metabolism of glutamate to glutamine by GS in the human brain.

Although the question as to whether ammonia in the brain can diffuse into the blood has previously been debated [35], back-flux of [^13^N]ammonia from brain to blood has now been demonstrated in healthy volunteers as well as subjects with cirrhosis [11, 19, 20]. Similar to these studies, our finding of small but positive values for [^13^N]ammonia *k*_2_ also indicate nonzero back-flux of ^13^N to blood. Consistent with this, the simplest irreversible model with a single tissue compartment and one rate constant, *K*_1_, showed a poor fit in nearly every dataset. The presence of non-negligible wash-out was also consistent with the plateau of the decay-corrected brain time-activity curves in conjunction with approximately 10% of parent tracer compound remaining in the arterial plasma at the end of the scan. Patlak plots were nonlinear at late times, also indicating the presence of reversibility of the tracer. Our data as well as that of Goldbecker et al. [20] indicate that back-flux of ammonia may be observed (although not reliably quantified) within 30 min of [^13^N]ammonia injection. Potential biochemical explanations for washout could include the immediate back-flux of ammonia as well as a longer-term action of glutaminase recycling ^13^N from the neuron back to the astrocyte [36].

The candidate compartmental models considered for [^13^N]ammonia in this study assumed that no ^13^N labelled metabolites crossed the blood brain barrier and contributed to brain tissue signal. We did not test this assumption explicitly, however, metabolites entering the brain would require a more complex kinetic model and would likely make the estimation of *k*_3_ in the parent [^13^N]ammonia component even less reliable due to increased numbers of parameters.

The process used in this study to separate parent [^13^N]ammonia from ^13^N labelled metabolites was taken from the method described by Keiding et al. [11]. However, over- or underestimated parent fractions from any uncertainties in this method and fitted model would affect the parent plasma input function and therefore the kinetic analysis. The model comparisons favoured a one-tissue compartment. If the parent fraction model overestimated the true plasma [^13^N]ammonia concentration, a positive *k*_2_ could be a bias to fit the data better and *k*_3_ even less likely separable from *K*_1_. An underestimate of parent plasma fraction would likely consider *k*_2_ an underestimate of true washout, though again is unlikely to improve *k*_3_ estimates.

Although *k*_2_ ICC values were high in this study, the parameter estimates correspond to a half-live of approximately 100 min and accurate parameter estimates would typically require longer scan durations and consequently isotopes with more suitable half-lives. Though dependent on noise levels and simulated model specifics, Turkheimer et al. [39] found the slowest identifiable components close to those corresponding to scan duration. Therefore we would not consider these values to be truly representative without further work.

While our data did not support estimation of GS activity, it did indicate that [^13^N]ammonia PET may provide an index of CBF. [^13^N]ammonia *K*_1_ correlated with the *K*_1_ (CBF) from the preceding [^15^O]water scans in the same subjects, and based on fractional difference metrics, [^13^N]ammonia brain uptake (*K*_1_) was of comparable reproducibility to [^15^O]water CBF measures over the test and retest scanning sessions. The correlations between *K*_1_ and CBF were observed when *K*_1_ was calculated with either a reversible one-tissue or reversible two-tissue compartment model, and to a lesser extent between *K*_*i*_ (calculated using Patlak analysis) and CBF. The correlations between *K*_1_ and CBF were qualitatively tighter for *K*_1_ calculated from the one compared to two-tissue compartmental model, as would be expected given there are fewer parameters. Nonetheless the slope of the best fit was not identical between subjects and scans (Fig. 3). Correlating CBF and *K*_1_ within region, across subjects and scans, did not yield significant correlations. In rhesus monkeys, Phelps et al. [37] found nonlinear relationships between *K*_1_ (or specifically extraction fraction, EF) and CBF, over a wide range of CBF values. Our data indicate that a linear relationship between *K*_1_ and CBF exists when CBF lies within the normal range investigated here.

Compared to the previous study of Keiding et al. [11] the values for [^13^N]ammonia *K*_1_ obtained in our study are approximately 35% lower and approximately 20% lower for CBF, though relative *K*_1_ and CBF between basal ganglia (putamen and thalamus), cerebellum and cortex were similar. In addition, inter-subject variances of both parameters were comparable to that study. The absolute values of CBF were also in keeping with the variances seen between centres for quantitative PET studies (approximately 40–60 mL/min 100g), as well as inter-scan and inter-subject variability (approximately 10%) [38]. The estimates for permeability-surface area product values and Flux_met_ were similar, while PS_met_ estimates were also slightly lower in our study, which is consistent with lower *K*_met_ found from the possibly unsuitable graphical method. As in Keiding et al., [11] we applied the Patlak method [32] to calculating *K*_met_ which avoided replicating the poor identification of *k*_2_ and *k*_3_ values with an explicit calculation. Nonetheless the fractional differences for *K*_met_, PS_met_ and Flux_met_ between test and retest scans were high.

This work uses standard PET acquisition and analysis methodology, however the rapid incorporation of ammonia into glutamine [29] along with the practicalities of using short half-life tracers (2.03 min and 9.97 min for ^15^O and ^13^N, respectively) and multiple scans with blood sampling within one day present challenges to accurately measuring the tracer kinetics which could have impacted parameter identifiability, model selection and repeatability. A number are explored here in more detail.

Measured blood curves would be delayed and dispersed between the arterial sampling point and the PET signal (further exaggerated by the length of the line from the arterial cannula to the Allogg detector). CBF was calculated with delay and dispersion explicitly included in the model fits [27], though standard 1TCM and 2TCM were explored for [^13^N]ammonia kinetics without this accounted for. As noted in work by Toussaint and Meyer [40] delay and dispersion estimates can be highly correlated, so while including these parameters improves accuracy of CBF, their individual estimates can be inaccurate. We did extend the 5-parameter Meyer model for CBF up to a 7-parameter 2TCM (i.e. including *k*_3_, *k*_4_, delay and dispersion) and applied this to the [^13^N]ammonia data analysis, however this did not improve *k*_3_ estimates and the preferred model remained the reversible 1TCM. In addition, increasing the number of free parameters increased the correlation and errors in parameter estimates.

Despite the preliminary work from simulations and dry runs, due to the blood processing time and to avoid low counts from ^13^N decay in subsequent scans, blood sample timings for [^13^N]ammonia scans were changed after the first subject’s scans (see Fig. 1, blue circles and lines) from 5-min intervals to those described in the methods. However the parent fraction fit is well within those of the remaining subjects used for the main analysis and those found by Keiding et al. [11] so we believe it is reasonable to assume this impact would be negligible.

Two errors in acquisition for some scans could have impacted the repeatability in CBF and/or *K*_1_. Subjects 1 and 2 did not have manual blood samples measured for the [^15^O]water scans. For these subjects, the average plasma-over-blood values from all remaining subjects were used (mean 1.14, s.d. 0.03). For the 2nd pair of scans for subject 3 and all 4 scans for subject 4, equipment failure meant that the exact timing of the continuous blood sampler was not recorded directly but was estimated from unsynced system logs after assessment of complete datasets. As an example, for ^15^O, a 20 s delay would result in a 12% bias from incorrect decay correction (2% for ^13^N). Both these errors (plasma and sampler timing) would effect the scaling of the plasma input functions but not other properties, hence there may be biases in CBF and *K*_1_ for these subjects, but not other rate-constants or model selection.

The parent fraction model was taken from the work of Keiding et al. [11], however the earliest blood sample was taken 4 min after tracer injection by which point most subjects showed a parent fraction under 50% (Fig. 1). It is possible that the biexponential fit is not ideal at earlier times and could influence the shape of the parent plasma curve, which may be necessary to accurately capture rapid kinetics. Similarly rapidly changing plasma-over-blood ratios (Additional file 1: Figure S2) would also have a similar impact. However, with standard PET methodology, continuous arterial sampling, while providing a detailed trace of the arterial concentration, does limit the ability to acquire large enough manual samples for further analysis without impacting the measurement of the blood peak.

The parent fraction estimation was also complicated by the outlying data from subjects 6, 7 and 8 (Fig. 1: black symbols and lines) in terms of values comparative to the remaining subjects or Keiding’s [11] data quality as well a quality of fit to the biexponential model. Smaller sample size does not just limit power but may also increase the impact of outliers. For the main analysis we considered data from the first 5 subjects only, however the analysis from all subjects did not improve repeatability (Additional file 1: Tables S8 and S9) or model selection. A population parent fraction curve from the first 5 subjects was also used to for model fits but neither did this improve repeatability or model selection for 2TCM.

Repeatability may have been limited by participant fatigue during the second scanning session, due to the technical complexity of the study and as subjects remained in the PET centre for an average of 3.5 h between the start of the first scan and end of the last. However, interscan variability for CBF and *K*_1_ were comparable and in line with previous CBF studies [38].

The strong correlations observed between *K*_1_ and CBF yielded variable slopes between subjects and scan pairs. It is unclear how much is attributable to a true representation of physiology or to unforeseen errors from the challenges of complex timing with short half-life tracers.

While the CBF and *K*_1_ ([^13^N]ammonia) correlated well within each scan pair, this study did not investigate the quantification or detectability of changes in perfusion per se. However, PET tracers with longer radioisotope half-lives are in general easier to incorporate into a scanning schedule and the slower washout of [^13^N]ammonia may yield images with improved signal to noise compared to [^15^O]water. Our results therefore suggest that the use of [^13^N]ammonia as a brain perfusion marker for low to normal blood flow may warrant further investigation.

## Conclusions

Based on our data, cerebral glutamate synthetase activity is not quantifiable using [^13^N]ammonia PET in healthy volunteers. Over a 30-min uptake period, tissue wash-out of the tracer is identifiable with a simple one-tissue compartmental model, suggesting irreversible models are not strictly appropriate. While the most repeatable uptake parameter, *K*_1_, would likely be a macroparameter combining perfusion, PS product and GS activity rather than assessing GS activity per se, it appears to correlate well with CBF and may be useful in this context.

## Supplementary information

**Additional file 1**. Supplementary Information.

## Data Availability

The datasets used during the current study are available from the corresponding author on reasonable request.
