# Supplementary material for: Evaluation of [13N]ammonia positron emission tomography as a potential method for quantifying glutamine synthetase activity in the human brain
Source: EJNMMI Res. 2020 Dec 3;10:146. doi: 10.1186/s13550-020-00731-0 (PMC7714883; doi:10.1186/s13550-020-00731-0)
Supplement: Supplementary file 1 — Additional file 1. Supplementary Information. [file 13550_2020_731_MOESM1_ESM.docx]

|  | N cases/controls | GS measure | Result |
| --- | --- | --- | --- |
| ***Schizophrenia*** | | | |
| Gluck et al., 2002 [1] | 27/13 | GS activity | No change, “DLPFC” (BA46) |
| Burbaeva et al., 2003 [2] | 8/9 | Protein | Decrease in “PFC” (BA10) |
| Prabakaran et al., 2004 [3] | 10/10 | Protein | Decrease in “PFC” (BA9) |
| Bruneau et al., 2005 [4] | 13/8 | mRNA | Increase in thalamus |
| Toro et al., 2006 [5] | 15/15 | Protein | Unchanged “PFC” (BA9) and “OFC” (BA11/47) |
| Burbaeva et al., 2007 [6] | 23/22 | Protein | Increase in “PFC”, cerebellum, no change in caudate nucleus. |
| Steffek et al., 2008 [7] | 23/27 | Protein | Decrease in “ACC” and STG, unchanged “DLPFC”, PVC, HC |
| Martins de Souza et al., 2010 [8] | 11/8 | Protein | Increase “ACC” |
| Katsel et al., 2011 [9] | 18/21 | mRNA | “ACC” (cingulate gyrus, BA24/32): no difference in layers I–III or underlying white matter; decrease in layers IV–VI. |
| Zhang et al., 2020 [10] | 35/34 | mRNA | Increase in “DLPFC” (BA46), no difference in “ACC (BA24)” |
| ***Depression and / or suicide*** | | | |
| Choudary et al., 2005 [11] | 9/7 | mRNA | Decrease “ACC” (BA24) and “DLPFC” (BA9/46) |
| Toro et al. 2006 [5] | 15/15 | Protein | Unchanged “PFC” (BA9) and “OFC” (BA11/47) |
| Kim et al., 2007 [12] |  | mRNA | “PFC” (BA10/46): Within schizophrenia, decreased in suicide (n=10) vs. non-suicide (n=35); within bipolar disorder, no difference in suicide (n=22) vs. non-suicide (n=23). |
| Karolewicz et al., 2009 [13] | 14/14 | Protein | No change amygdala |
| Sequeira et al., 2009 [14] | <20, varying by region. | mRNA | Of 17 regions analysed, decrease in “DLPFC” in BA44 BA45, BA46, and in amygdala. |
| Klemplan et al., 2009 [15] | 24/13 | mRNA | Decrease in “ventral PFC“ (BA44, 45, 46, 47) |
| Kekesi et al., 2012 [16] | 6/6 | Protein | Increase in “PFC”, no difference in amygdala. |
| Chandley et al., 2013 [17] | 19/20 | mRNA | Decreased in pontine LC homogenates, no difference in “PFC” (BA10) |
| Zhao et al., 2016 [18] | 17 MDD-S  7 MDD-NS  12-Con | mRNA | Decreased in MDD+suicide compared to MDD non-suicide in “DLPFC” (BA46), no difference in “ACC” (BA24). |
| Zhang et al., 2020 [10] | 17/7 | mRNA | Increased in schizophrenia suicide completers (n=17) compared to schizophrenia non-suicide (n=7) in “DLPFC” (BA46), no difference in “ACC” (BA24) |
| ***Bipolar disorder*** | | | |
| Toro et al. 2006 [5] | 15/15 | Protein | Unchanged “PFC” (BA9) and “OFC” (BA11/47) |
| Choudary et al., 2005 [11] | 6/7 | mRNA | Unchanged in “ACC” (BA24) and “DLPFC” (BA9/46) |
| Bernard et al 2011 [19] | 6/9 | mRNA | Unchanged in LC |
| ***Autism Spectrum Disorder*** | | | |
| Shimmura et al., 2013 [20] | 7/13 | Protein | Unchanged “ACC” |
| ***Temporal Lobe Epilepsy (TLE)*** | | | |
| van der Hel et al., 2005 [21] |  | Protein and activity | decreased in TLE with sclerosis hippocampus (CA1, CA4) |
| Eid et al., 2013 [22] | Autopsy 3  MTLE 9  Non-MTLE 6 | mRNA | Increased in CA3 (but not CA1, CA2, subiculum, dentate gyrus or hilus) in non-MTLE CA3 |
| ***Alcohol use disorder*** | | | |
| Matsuda-Matsumoto et al., 2007 [23] | 6/8 | Protein | Decrease HC |
| Miguel-Hilgado et al., 2010 [24] | 13/13 | Protein | No change “OFC” (BA47) |
| ***Huntington’s Disease*** | | | |
| Carter et al., 1982 [25] | 15-21/15-20 | GS activity | Decreased in frontal, temporal cortex, putamen, cerebellum. Unchanged thalamus, hippocampus, olivary nucleus |
| ***Alzheimer’s Disease*** | | | |
| Gluck et al., 2002 [1] | 10/13 | GS activity | No change “DLPFC” (BA46) |
| Burbaeva et al., 2005 [26] | 11/11 | Protein | Increased “PFC” (BA10) |
| ***Hepatic encephalopathy*** | | | |
| Lavoie et al., 1987 [27] | 9/9 | GS activity | Decreased caudate. |

**Supplementary Table 1**. Post-mortem studies of glutamine synthetase (GS, also known as glutamate-ammonia ligase) in psychiatric or neurological disorders. N c/c: number of cases / controls. MTLE: temporal lobe epilepsy with hippocampal sclerosis; non-MTLE: TLE without hippocampal sclerosis. Region abbreviations: ACC: anterior cingulate cortex; BA Brodmann area; “DLPFC”: dorsolateral “prefrontal” cortex; HC: hippocampus; LC: locus coeruleus; OFC: orbitofrontal cortex: PFC: “prefrontal” cortex; PVC: primary visual cortex; STG: superior temporal gyrus

glutamine synthetase

glutamate

k_4_

k_3_

k_2_

K_1_

CBF

BBB

Brain tissue

Capillary

[^13^N]glutamine

[^13^N]NH_3_

[^13^N]NH_3_ uptake

[^13^N]N H_3_ washout

C_1_

C_2_

**Supplementary Figure 1.** **Two tissue [^13^N]NH_3_ Compartmental Model (simplified model based on Cooper 2011 [28]).**

K_1_, (mL blood/ min/mL tissue) is the rate constant of initial uptake (or “blood clearance”) of [^13^N]NH_3_ from the blood to brain tissue (compartment 1, C_1_) across the blood brain barrier. This parameter would be expected to be a function of blood flow and capillary permeability. k_2_ (1/min) is the rate constant of backflux of [^13^N]NH_3_ from brain to blood (1/min). The rate constant k_3_ (1/min) represents the conversion by glutamine synthetase of [^13^N]NH_3_ and endogenous glutamate to [^13^N]glutamine (compartment C_2_). k_4_ (1/min) is the rate constant representing the recycling of [^13^N]glutamine to [^13^N]glutamate and [^13^N]NH_3_ by glutaminase and glutamate dehydrogenase. This route would be considered negligible in the irreversible two tissue compartment model considered by Keiding et al. [29] Further simplification of this model (i.e. reversible and irreversible one tissue compartment model) would assume either rapid equilibration of C_1_ and C_2_, or k_3_ ~ 0, with remaining rate constants representing a mixture of the processes described.


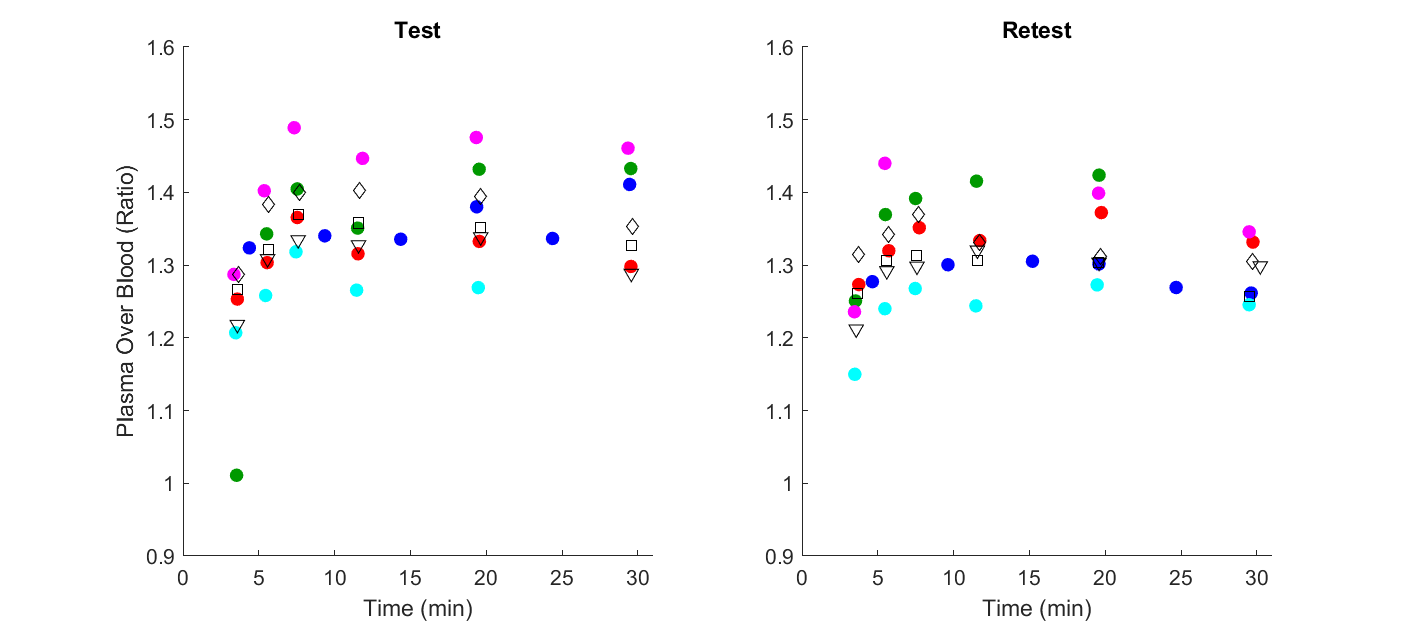


**Supplementary Figure 2: Plasma Over Blood Ratio**

Plasma over blood (POB) ratio data from test scan (left) and retest scan (right) for each subject. Symbols represent ratio of plasma concentration to whole arterial blood concentration measured from individual arterial samples. Circles show colour-coded subjects 1-5 used in the full analysis (blue, green, red, magenta & cyan, for subjects 1-5 respectively). Black symbols (triangle, square, diamond for subjects 6-8 respectively) show subjects 6-8 (not included in main analysis). Mean POB value was calculated for each subject and scan and used for the blood data processing. Haematocrit (HCT) was not measured for subjects, though since tracer is injected into plasma space it would be expected that at t=0, true POB = 1/(1-HCT) (approximately 1.6 for healthy subjects).


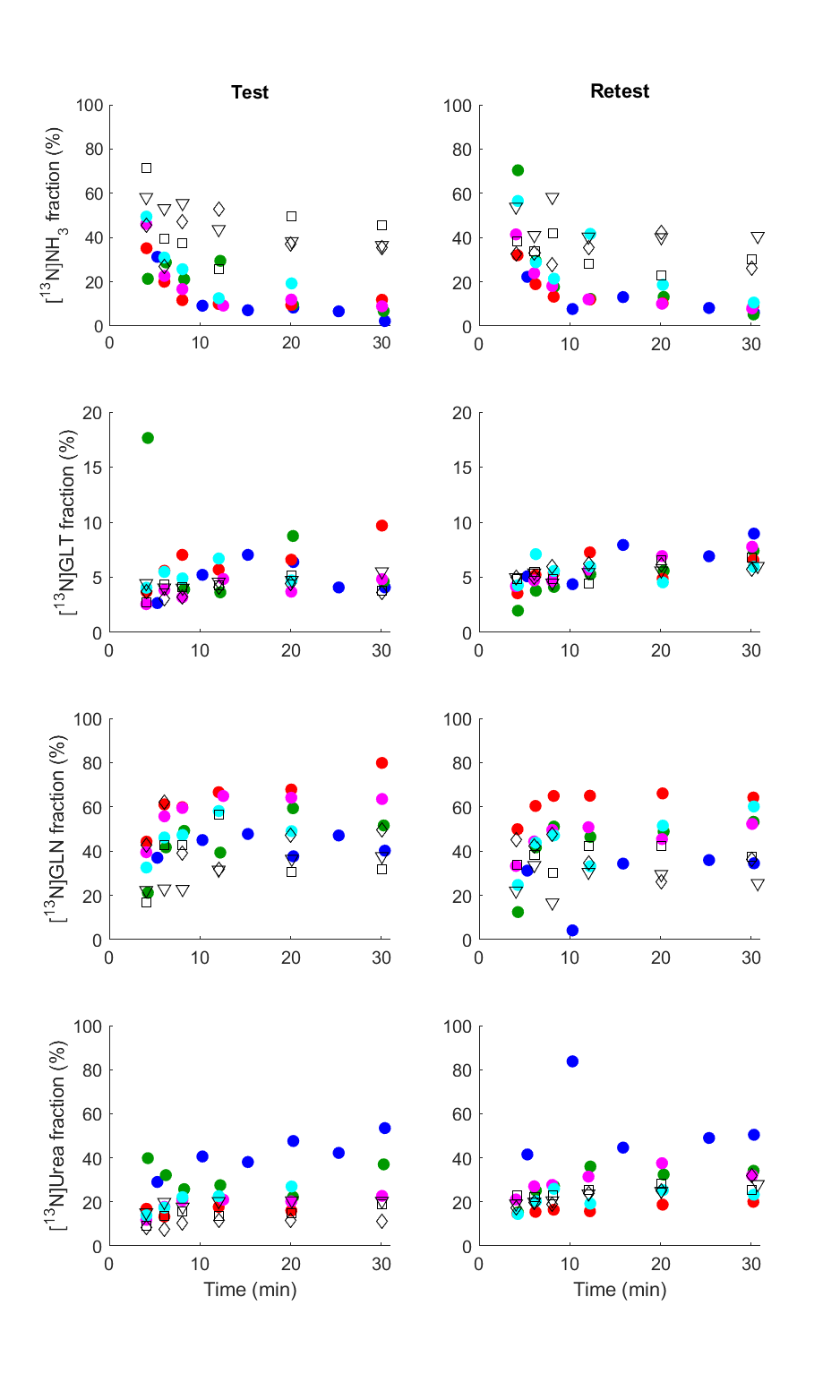


**Supplementary Figure 3: Separated ^13^N labelled compound plasma fractions**

Plots showing subject data for separated ^13^N labelled compounds measured from metabolite separation analysis from each manual plasma sample. Fraction for each compound is calculated from the total of all. 1^st^ column: test scan data; 2^nd^ column retest scan data. 1^st^ row: [^13^N]ammonia fraction; 2^nd^ row: [^13^N]glutamate fraction; 3^rd^ row: [^13^N]glutamine; 4^th^ row: [^13^N]urea. Circles show colour-coded subjects 1-5 used in the full analysis (blue, green, red, magenta & cyan, for subjects 1-5 respectively). Black symbols (triangle, square, diamond for subjects 6-8 respectively) show subjects 6-8 (not included in main analysis). Only the parent compound, [^13^N]ammonia, was required to be fitted to the biexponential function to calculate the plasma parent input function.

| ROI | PSbbb (ml/min/100g) | | Extraction Fraction | | Kmet (ml/min/100g) | | PSmet (ml/min/100g) | | Flux_met (nmol/min/100g) | |
| --- | --- | --- | --- | --- | --- | --- | --- | --- | --- | --- |
|  | S1 | S2 | S1 | S2 | S1 | S2 | S1 | S2 | S1 | S2 |
| Grey Matter | 26.8 ± 2.8 | 25.4 ± 2.4 | 0.45 ± 0.03 | 0.47 ± 0.03 | 12.3 ± 3.7 | 11.5 ± 1.7 | 14.6 ± 5.0 | 13.6 ± 2.4 | 319 ± 95 | 332 ± 50 |
| White Matter | 20.6 ± 2.5 | 19.4 ± 1.5 | 0.52 ± 0.04 | 0.53 ± 0.03 | 10.0 ± 2.6 | 9.3 ± 1.0 | 12.5 ± 4.0 | 11.6 ± 1.6 | 259 ± 68 | 271 ± 30 |
| Hippocampus_l | 30.2 ± 7.1 | 28.3 ± 4.0 | 0.59 ± 0.08 | 0.58 ± 0.05 | 12.3 ± 2.1 | 8.0 ± 1.8 | 15.5 ± 3.1 | 9.3 ± 2.4 | 320 ± 54 | 233 ± 51 |
| OL_ling_G_l | 34.1 ± 6.8 | 31.1 ± 1.9 | 0.53 ± 0.07 | 0.54 ± 0.02 | 13.4 ± 4.5 | 13.6 ± 2.3 | 16.3 ± 6.6 | 16.7 ± 3.4 | 349 ± 116 | 394 ± 66 |
| G_cing_post_l | 29.7 ± 3.0 | 28.3 ± 2.4 | 0.46 ± 0.03 | 0.48 ± 0.03 | 14.0 ± 4.8 | 11.9 ± 1.5 | 17.0 ± 6.9 | 14.0 ± 2.1 | 365 ± 125 | 346 ± 43 |
| Putamen_l | 31.5 ± 3.6 | 28.4 ± 2.8 | 0.47 ± 0.04 | 0.46 ± 0.03 | 14.4 ± 5.6 | 13.0 ± 1.7 | 17.5 ± 7.3 | 15.3 ± 2.3 | 375 ± 146 | 376 ± 48 |
| Thalamus_l | 30.6 ± 4.5 | 28.5 ± 2.2 | 0.46 ± 0.05 | 0.46 ± 0.03 | 13.1 ± 3.7 | 12.9 ± 1.4 | 15.5 ± 5.1 | 15.1 ± 2.0 | 342 ± 97 | 373 ± 42 |
| Cerebellum_l | 29.8 ± 3.7 | 27.7 ± 2.4 | 0.44 ± 0.04 | 0.46 ± 0.03 | 14.5 ± 3.3 | 12.6 ± 2.3 | 17.3 ± 4.7 | 14.8 ± 3.2 | 378 ± 86 | 364 ± 67 |

**Supplementary Table 2:** **Permeability–surface area and fractional uptake rate parameters calculated from [^13^N]ammonia model parameters from subjects 1-5.**

The table provides model parameters for test (S1) & retest (S2) scans for grey matter and selected regions of interest (ROIs). The table presents estimates of PS_BBB_ (flow independent permeability-surface area product of the blood brain barrier to [^13^N]ammonia); Extraction Fraction (K_1_/CBF); Kmet (net metabolic clearance of [^13^N]ammonia in blood into intracellular [^13^N]glutamine), PSmet (flow-independent permeability-surface area product of conversion of ammonia to intracellular glutamine) and CMRA (cerebral metabolic rate of ammonia). Data are presented as mean ± standard deviation for grey matter, white matter and the left (l) hippocampus, lingual gyrus (OL_ling_G), posterior cingulate gyrus (G_cing_post), putamen, thalamus, and cerebellum. Reported for comparison with results from work of Keiding et al (2006) [29].

| ROI | PS_bbb_ (ml/min/100g) | | | Extraction Fraction | | | K_met_ (ml/min/100g) | | | PS_met_ (ml/min/100g) | | | Flux_met_ (nmol/min/100g) | | |
| --- | --- | --- | --- | --- | --- | --- | --- | --- | --- | --- | --- | --- | --- | --- | --- |
|  | ICC | Var | AbsVar | ICC | Var | AbsVar | ICC | Var | AbsVar | ICC | Var | AbsVar | ICC | Var | AbsVar |
| Grey Matter | -0.78 | -5.0 ± 18.5 | 12.9 ± 12.8 | -0.8 | 5.2 ± 13.3 | 11.8 ± 6.2 | 0.58 | -4.0 ± 23.2 | 17.7 ± 13.0 | 0.6 | -3.1 ± 26.7 | 20.2 ± 14.7 | 0.62 | 6.8 ± 23.1 | 16.2 ± 16.2 |
| White Matter | -0.87 | -5.3 ± 19.1 | 13.6 ± 13.0 | -0.93 | 2.3 ± 12.9 | 10.4 ± 6.3 | 0.57 | -3.9 ± 20.1 | 15.2 ± 11.5 | 0.58 | -3.6 ± 24.4 | 17.5 ± 15.1 | 0.61 | 6.9 ± 20.0 | 14.7 ± 13.8 |
| Hippocampus_l | 0.32 | -5.2 ± 21.1 | 17.1 ± 10.8 | 0.36 | -0.4 ± 12.9 | 11.0 ± 3.9 | 0.27 | -42.1 ± 23.2 | 42.1 ± 23.2 | 0.26 | -49.3 ± 26.5 | 49.3 ± 26.5 | 0.27 | -31.6 ± 23.8 | 31.6 ± 23.8 |
| OL_ling_G_l | -0.4 | -7.7 ± 23.3 | 16.2 ± 16.9 | -0.46 | 2.2 ± 15.3 | 12.2 ± 7.4 | 0.71 | 4.8 ± 21.8 | 13.2 ± 16.9 | 0.73 | 7.1 ± 25.3 | 15.8 ± 19.7 | 0.76 | 15.5 ± 21.6 | 18.8 ± 18.0 |
| G_cing_post_l | -0.79 | -4.6 ± 17.7 | 13.0 ± 11.4 | -0.8 | 5.0 ± 12.8 | 11.1 ± 6.6 | 0.49 | -11.9 ± 25.8 | 23.4 ± 12.6 | 0.48 | -13.0 ± 30.4 | 27.2 ± 14.4 | 0.54 | -1.1 ± 25.8 | 21.2 ± 10.2 |
| Putamen_l | -0.82 | -10.2 ± 20.1 | 16.1 ± 14.4 | -0.85 | -2.0 ± 14.5 | 11.2 ± 7.8 | 0.38 | -2.0 ± 47.8 | 35.2 ± 27.2 | 0.41 | -3.2 ± 52.5 | 39.2 ± 29.1 | 0.42 | 8.4 ± 46.9 | 28.3 ± 35.9 |
| Thalamus_l | -0.63 | -6.3 ± 20.7 | 15.6 ± 13.2 | -0.67 | 1.3 ± 14.9 | 12.4 ± 5.7 | 0.47 | 0.8 ± 22.7 | 18.2 ± 10.0 | 0.48 | 1.7 ± 26.3 | 21.5 ± 10.9 | 0.51 | 11.6 ± 22.6 | 20.4 ± 12.7 |
| Cerebellum_l | -0.56 | -7.0 ± 18.3 | 12.3 ± 14.5 | -0.6 | 2.9 ± 13.3 | 10.6 ± 6.9 | 0.71 | -13.9 ± 16.6 | 16.4 ± 13.3 | 0.72 | -14.6 ± 19.2 | 18.9 ± 13.8 | 0.73 | -3.0 ± 16.6 | 14.3 ± 5.7 |

**Supplementary Table 3:** **Repeatability for Permeability–surface area and fractional uptake rate parameters calculated from [^13^N]ammonia model parameters from subjects 1-5.**

| ROI | CBF (ml/min/100g) | | k’_2_ (1/min) | | Delay (s) | | Dispersion (1/s) | | Vb | |
| --- | --- | --- | --- | --- | --- | --- | --- | --- | --- | --- |
|  | S1 | S2 | S1 | S2 | S1 | S2 | S1 | S2 | S1 | S2 |
| Grey Matter | 37.6 ± 4.8 | 38.5 ± 3.4 | 0.541 ± 0.063 | 0.560 ± 0.036 | 1.18 ± 1.71 | 0.93 ± 1.07 | 7.6 ± 4.4 | 5.9 ± 3.5 | 0.082 ± 0.047 | 0.095 ± 0.043 |
| White Matter | 25.8 ± 3.4 | 26.4 ± 2.7 | 0.402 ± 0.045 | 0.411 ± 0.033 | 1.55 ± 2.43 | 1.44 ± 1.20 | 4.9 ± 4.1 | 4.0 ± 4.0 | 0.074 ± 0.028 | 0.078 ± 0.030 |
| Hippocampus_l | 32.1 ± 3.7 | 33.8 ± 3.1 | 0.406 ± 0.062 | 0.430 ± 0.038 | 1.39 ± 1.60 | 2.35 ± 2.49 | 6.2 ± 2.3 | 5.1 ± 3.4 | 0.077 ± 0.032 | 0.071 ± 0.028 |
| OL_ling_G_l | 40.8 ± 5.4 | 41.5 ± 2.8 | 0.559 ± 0.084 | 0.571 ± 0.043 | 0.90 ± 1.77 | 0.76 ± 1.40 | 7.4 ± 3.9 | 6.2 ± 4.0 | 0.091 ± 0.052 | 0.100 ± 0.053 |
| G_cing_post_l | 43.6 ± 5.3 | 44.3 ± 4.2 | 0.584 ± 0.069 | 0.604 ± 0.042 | 0.92 ± 1.75 | 0.78 ± 1.13 | 6.9 ± 4.6 | 6.4 ± 5.4 | 0.109 ± 0.057 | 0.109 ± 0.061 |
| Putamen_l | 45.3 ± 7.3 | 46.1 ± 5.5 | 0.575 ± 0.096 | 0.601 ± 0.071 | 0.84 ± 1.10 | 1.41 ± 1.63 | 9.2 ± 4.5 | 8.9 ± 4.3 | 0.081 ± 0.042 | 0.072 ± 0.055 |
| Thalamus_l | 43.9 ± 5.5 | 44.5 ± 4.2 | 0.589 ± 0.078 | 0.609 ± 0.052 | 0.01 ± 0.85 | 0.52 ± 1.27 | 9.5 ± 3.1 | 9.5 ± 4.9 | 0.073 ± 0.035 | 0.074 ± 0.059 |
| Cerebellum_l | 41.7 ± 6.8 | 42.6 ± 5.2 | 0.538 ± 0.084 | 0.553 ± 0.057 | 0.49 ± 1.71 | 0.76 ± 1.08 | 8.5 ± 3.3 | 6.9 ± 5.1 | 0.073 ± 0.037 | 0.080 ± 0.045 |

**Supplementary Table 4: Full parameter estimates (between-subject mean +/- s.d.) for [^15^O]water data modelling.**

| ROI | CBF (ml/min/100g) | | | k’_2_ (1/min) | | | Delay (s) | | | Dispersion (1/s) | | | Vb | | |
| --- | --- | --- | --- | --- | --- | --- | --- | --- | --- | --- | --- | --- | --- | --- | --- |
|  | ICC | Var | AbsVar | ICC | Var | AbsVar | ICC | Var | AbsVar | ICC | Var | AbsVar | ICC | Var | AbsVar |
| Grey Matter | 0.5 | 3 ± 11 | 9 ± 6 | 0.65 | 4 ± 8 | 7 ± 6 | 0.38 | 38 ± 141 | 80 ± 119 | 0.39 | -34 ± 72 | 67 ± 37 | 0.82 | 19 ± 40 | 33 ± 28 |
| White Matter | 0.69 | 2 ± 10 | 7 ± 6 | 0.84 | 2 ± 6 | 5 ± 4 | 0.24 | 56 ± 168 | 53 ± 169 | 0.32 | -42 ± 128 | 111 ± 66 | 0.61 | 1 ± 55 | 32 ± 42 |
| Hippocampus_l | 0.17 | 5 ± 13 | 11 ± 9 | 0.6 | 6 ± 12 | 10 ± 8 | 0.57 | 32 ± 128 | 73 ± 107 | 0.33 | -30 ± 77 | 65 ± 47 | 0.59 | -8 ± 44 | 35 ± 25 |
| OL_ling_G_l | 0.22 | 2 ± 13 | 10 ± 9 | 0.49 | 3 ± 13 | 9 ± 8 | 0.4 | 71 ± 182 | 73 ± 181 | 0.31 | -37 ± 106 | 80 ± 75 | 0.74 | 16 ± 53 | 44 ± 29 |
| G_cing_post_l | 0.68 | 2 ± 9 | 8 ± 4 | 0.72 | 4 ± 8 | 7 ± 5 | 0.63 | -401 ± 907 | -304 ± 949 | 0.51 | -28 ± 101 | 72 ± 71 | 0.76 | -2 ± 50 | 38 ± 30 |
| Putamen_l | 0.85 | 2 ± 8 | 7 ± 2 | 0.88 | 5 ± 8 | 7 ± 5 | 0.38 | 5 ± 179 | 50 ± 171 | -0.09 | 1 ± 82 | 64 ± 44 | 0.36 | -23 ± 95 | 71 ± 61 |
| Thalamus_l | 0.66 | 2 ± 9 | 7 ± 5 | 0.7 | 4 ± 8 | 7 ± 5 | 0.23 | -8 ± 183 | 92 ± 154 | 0.4 | -8 ± 63 | 45 ± 42 | 0.62 | -24 ± 69 | 54 ± 47 |
| Cerebellum_l | 0.69 | 3 ± 12 | 9 ± 7 | 0.77 | 3 ± 10 | 8 ± 6 | 0.56 | 157 ± 379 | 197 ± 357 | 0.48 | -42 ± 76 | 59 ± 62 | 0.61 | 3 ± 67 | 49 ± 42 |

**Supplementary Table 5:** **Full repeatability statistics of parameter estimates (between-subject mean +/- s.d.) for [^15^O]water data modelling.**

| ROI | K1 (ml/min/100g) | | k2 (1/min) | | k3 (1/min) | | Vb | |
| --- | --- | --- | --- | --- | --- | --- | --- | --- |
|  | S1 | S2 | S1 | S2 | S1 | S2 | S1 | S2 |
| Grey Matter | 20.1 ± 1.5 | 18.7 ± 1.3 | 0.0072 ± 0.0035 | 0.0096 ± 0.0042 | 0.0031 ± 0.0045 | 0.0177 ± 0.0259 | 0.089 ± 0.012 | 0.078 ± 0.006 |
| White Matter | 14.6 ± 1.1 | 13.7 ± 0.7 | 0.0073 ± 0.0043 | 0.0096 ± 0.0053 | 0.0139 ± 0.0177 | 0.0274 ± 0.0401 | 0.060 ± 0.008 | 0.052 ± 0.003 |
| Hippocampus_l | 19.7 ± 2.8 | 18.8 ± 1.6 | 0.0256 ± 0.0145 | 0.0360 ± 0.0212 | 0.0532 ± 0.0261 | 0.0621 ± 0.0304 | 0.086 ± 0.011 | 0.074 ± 0.009 |
| OL_ling_G_l | 23.7 ± 3.0 | 21.6 ± 0.9 | 0.0072 ± 0.0023 | 0.0090 ± 0.0035 | 0.0000 ± 0.0000 | 0.0125 ± 0.0172 | 0.100 ± 0.019 | 0.088 ± 0.013 |
| G_cing_post_l | 22.2 ± 1.6 | 20.8 ± 1.3 | 0.0069 ± 0.0038 | 0.0099 ± 0.0059 | 0.0038 ± 0.0052 | 0.0154 ± 0.0231 | 0.086 ± 0.013 | 0.077 ± 0.004 |
| Putamen_l | 23.3 ± 1.9 | 21.2 ± 1.5 | 0.0063 ± 0.0028 | 0.0086 ± 0.0037 | 0.0000 ± 0.0000 | 0.0144 ± 0.0203 | 0.084 ± 0.015 | 0.072 ± 0.007 |
| Thalamus_l | 22.8 ± 2.4 | 21.2 ± 1.2 | 0.0128 ± 0.0057 | 0.0178 ± 0.0075 | 0.0241 ± 0.0214 | 0.0405 ± 0.0234 | 0.091 ± 0.010 | 0.075 ± 0.004 |
| Cerebellum_l | 22.5 ± 2.0 | 20.7 ± 1.3 | 0.0070 ± 0.0047 | 0.0088 ± 0.0034 | 0.0056 ± 0.0126 | 0.0145 ± 0.0216 | 0.098 ± 0.019 | 0.085 ± 0.008 |

**Supplementary Table 6:** **Parameter estimates for subjects 1-5 (between-subject mean +/- s.d.) for [^13^N]ammonia data modelling, irreversible 2TCM.**

| ROI | K1 (ml/min/100g) | | | k2 (1/min) | | | k3 (1/min) | | | Vb | | |
| --- | --- | --- | --- | --- | --- | --- | --- | --- | --- | --- | --- | --- |
|  | ICC | Var | AbsVar | ICC | Var | AbsVar | ICC | Var | AbsVar | ICC | Var | AbsVar |
| Grey Matter | -0.79 | -7 ± 13 | 10 ± 11 | 0.94 | 29 ± 18 | 29 ± 18 | 0.02 | -30 ± 179 | 153 ± 62 | 0.05 | -13 ± 15 | 15 ± 11 |
| White Matter | -0.89 | -6 ± 13 | 9 ± 10 | 0.93 | 28 ± 18 | 28 ± 18 | -0.05 | -46 ± 163 | 141 ± 67 | -0.1 | -13 ± 16 | 17 ± 11 |
| Hippocampus_l | 0.35 | -4 ± 13 | 10 ± 8 | 0.62 | 34 ± 44 | 48 ± 23 | 0.22 | 15 ± 49 | 39 ± 27 | -0.43 | -15 ± 21 | 20 ± 15 |
| OL_ling_G_l | -0.42 | -8 ± 15 | 10 ± 14 | 0.86 | 21 ± 13 | 21 ± 13 | 0 | 71 ± 165 | 147 ± 80 | 0.79 | -13 ± 11 | 13 ± 10 |
| G_cing_post_l | -0.78 | -7 ± 13 | 9 ± 11 | 0.86 | 34 ± 24 | 34 ± 24 | 0.15 | 13 ± 196 | 173 ± 37 | -0.21 | -11 ± 18 | 15 ± 14 |
| Putamen_l | -0.84 | -9 ± 14 | 12 ± 12 | 0.85 | 31 ± 24 | 31 ± 24 | 0 | 59 ± 194 | 181 ± 33 | 0.22 | -15 ± 18 | 17 ± 15 |
| Thalamus_l | -0.64 | -7 ± 15 | 11 ± 11 | 0.76 | 35 ± 27 | 37 ± 25 | 0.42 | 80 ± 95 | 105 ± 57 | -0.29 | -19 ± 14 | 19 ± 14 |
| Cerebellum_l | -0.58 | -8 ± 13 | 9 ± 13 | 0.91 | 30 ± 27 | 32 ± 24 | 0.22 | -58 ± 157 | 138 ± 73 | 0.52 | -13 ± 15 | 16 ± 11 |

**Supplementary Table 7:** **Parameter estimate repeatability for subjects 1-5 (between-subject mean +/- s.d.) for [^13^N]ammonia data modelling, irreversible 2TCM.**

| ROI | K1 (ml/min/100g) | | k2 (1/min) | | k3 (1/min) | | Vb |  |
| --- | --- | --- | --- | --- | --- | --- | --- | --- |
|  | S1 | S2 | S1 | S2 | S1 | S2 | S1 | S2 |
| Grey Matter | 18.6 ± 2.4 | 21.6 ± 6.8 | 0.0154 ± 0.0124 | 0.0321 ± 0.0371 | 0.0037 ± 0.0056 | 0.0142 ± 0.0203 | 0.077 ± 0.019 | 0.072 ± 0.010 |
| White Matter | 14.1 ± 1.5 | 17.3 ± 8.5 | 0.0156 ± 0.0129 | 0.0332 ± 0.0388 | 0.0113 ± 0.0153 | 0.0214 ± 0.0314 | 0.054 ± 0.011 | 0.050 ± 0.005 |
| Hippocampus_l | 18.5 ± 2.7 | 22.1 ± 7.6 | 0.0332 ± 0.0197 | 0.0585 ± 0.0380 | 0.0401 ± 0.0290 | 0.0464 ± 0.0318 | 0.075 ± 0.017 | 0.070 ± 0.009 |
| OL_ling_G_l | 22.1 ± 3.1 | 26.2 ± 10.3 | 0.0152 ± 0.0118 | 0.0330 ± 0.0396 | 0.0012 ± 0.0033 | 0.0114 ± 0.0132 | 0.087 ± 0.024 | 0.080 ± 0.015 |
| G_cing_post_l | 21.3 ± 2.5 | 25.8 ± 12.3 | 0.0147 ± 0.0115 | 0.0321 ± 0.0372 | 0.0031 ± 0.0044 | 0.0123 ± 0.0181 | 0.077 ± 0.017 | 0.075 ± 0.007 |
| Putamen_l | 21.7 ± 2.9 | 25.2 ± 10.6 | 0.0150 ± 0.0131 | 0.0321 ± 0.0389 | 0.0021 ± 0.0057 | 0.0124 ± 0.0160 | 0.072 ± 0.020 | 0.065 ± 0.011 |
| Thalamus_l | 21.3 ± 2.8 | 25.4 ± 10.2 | 0.0203 ± 0.0125 | 0.0418 ± 0.0383 | 0.0171 ± 0.0195 | 0.0303 ± 0.0227 | 0.080 ± 0.018 | 0.070 ± 0.009 |
| Cerebellum_l | 20.8 ± 2.9 | 23.9 ± 7.2 | 0.0150 ± 0.0124 | 0.0311 ± 0.0370 | 0.0052 ± 0.0104 | 0.0119 ± 0.0169 | 0.085 ± 0.024 | 0.079 ± 0.012 |

**Supplementary Table 8:** **Parameter estimates for all 8 subjects (between-subject mean +/- s.d.) for [^13^N]ammonia data modelling, irreversible 2TCM.**

This data is shown for completeness though does include the data from the 3 subjects with outlying parent fractions. Most notably the outlying biexponential fit in the parent fraction for subject 8 (see Figure 1B, main manuscript) yielding comparatively large values of K_1_ (due to dramatically reduced peak in plasma parent input function).

| ROI | K1 (ml/min/100g) | |  | k2 (1/min) | |  | k3 (1/min) | |  | Vb |  |  |
| --- | --- | --- | --- | --- | --- | --- | --- | --- | --- | --- | --- | --- |
|  | ICC | Var | AbsVar | ICC | Var | AbsVar | ICC | Var | AbsVar | ICC | Var | AbsVar |
| Grey Matter | -0.4 | 11.8 ± 34.0 | 22.2 ± 27.4 | 0.41 | 45.1 ± 40.5 | 45.1 ± 40.5 | -0.05 | 15.5 ± 179.8 | 161.3 ± 53.6 | 0.61 | -4.8 ± 15.9 | 12.6 ± 9.9 |
| White Matter | 0.11 | 12.9 ± 33.8 | 22.7 ± 27.3 | 0.4 | 45.4 ± 42.0 | 45.4 ± 42.0 | -0.01 | 10.8 ± 170.4 | 148.6 ± 62.6 | 0.33 | -5.6 ± 16.6 | 13.1 ± 10.8 |
| Hippocampus_l | -0.25 | 13.8 ± 33.7 | 22.9 ± 27.5 | 0.29 | 47.2 ± 52.0 | 55.7 ± 41.3 | 0.54 | 33.4 ± 86.1 | 67.5 ± 59.1 | 0.37 | -6.2 ± 20.1 | 15.6 ± 13.1 |
| OL_ling_G_l | -0.24 | 12.0 ± 36.9 | 23.6 ± 29.8 | 0.39 | 42.1 ± 42.0 | 42.2 ± 41.9 | -0.05 | 92.2 ± 144.5 | 144.1 ± 82.8 | 0.83 | -6.0 ± 12.4 | 10.0 ± 9.1 |
| G_cing_post_l | 0.12 | 12.7 ± 33.2 | 22.6 ± 26.5 | 0.42 | 49.4 ± 39.0 | 49.4 ± 39.0 | 0.14 | 48.7 ± 177.9 | 167.6 ± 47.5 | 0.28 | -1.6 ± 20.1 | 14.5 ± 12.9 |
| Putamen_l | -0.07 | 10.0 ± 34.7 | 23.4 ± 26.4 | 0.4 | 46.4 ± 43.6 | 46.4 ± 43.6 | -0.17 | 63.7 ± 182.3 | 180.3 ± 26.9 | 0.64 | -7.8 ± 17.4 | 13.8 ± 12.4 |
| Thalamus_l | -0.13 | 13.0 ± 34.8 | 23.9 ± 27.5 | 0.36 | 51.4 ± 42.2 | 52.3 ± 40.9 | 0.55 | 93.7 ± 107.0 | 121.7 ± 67.6 | 0.57 | -10.6 ± 15.4 | 12.7 ± 13.5 |
| Cerebellum_l | -0.51 | 11.2 ± 34.9 | 21.7 ± 28.8 | 0.4 | 45.9 ± 43.0 | 47.1 ± 41.5 | 0.16 | -2.4 ± 174.4 | 152.4 ± 62.3 | 0.7 | -5.1 ± 16.0 | 13.0 ± 9.6 |

**Supplementary Table 9:** **Repeatability for parameter estimates for all 8 subjects (between-subject mean +/- s.d.) for [^13^N]ammonia data modelling, irreversible 2TCM.**

| ROI | K1 (ml/min/100g) | | k2 (1/min) | | Vb | | VT (ml/g) | |
| --- | --- | --- | --- | --- | --- | --- | --- | --- |
|  | S1 | S2 | S1 | S2 | S1 | S2 | S1 | S2 |
| Grey Matter | 20.1 ± 1.5 | 18.6 ± 1.3 | 0.0069 ± 0.0029 | 0.0074 ± 0.0022 | 0.089 ± 0.012 | 0.079 ± 0.007 | 33.1 ± 11.6 | 26.4 ± 5.9 |
| White Matter | 14.5 ± 1.2 | 13.5 ± 0.8 | 0.0059 ± 0.0027 | 0.0064 ± 0.0021 | 0.060 ± 0.008 | 0.053 ± 0.005 | 28.5 ± 11.3 | 22.5 ± 5.8 |
| Hippocampus_l | 18.9 ± 2.4 | 17.6 ± 1.0 | 0.0122 ± 0.0061 | 0.0144 ± 0.0041 | 0.092 ± 0.014 | 0.082 ± 0.012 | 18.3 ± 7.5 | 13.0 ± 3.5 |
| OL_ling_G_l | 23.7 ± 3.0 | 21.5 ± 1.0 | 0.0072 ± 0.0023 | 0.0075 ± 0.0020 | 0.100 ± 0.019 | 0.088 ± 0.013 | 35.4 ± 9.7 | 30.3 ± 7.2 |
| G_cing_post_l | 22.2 ± 1.6 | 20.6 ± 1.3 | 0.0065 ± 0.0032 | 0.0075 ± 0.0019 | 0.087 ± 0.013 | 0.078 ± 0.006 | 40.7 ± 19.0 | 28.8 ± 6.5 |
| Putamen_l | 23.3 ± 1.9 | 21.1 ± 1.5 | 0.0063 ± 0.0028 | 0.0072 ± 0.0028 | 0.084 ± 0.015 | 0.072 ± 0.008 | 41.9 ± 14.8 | 32.5 ± 10.8 |
| Thalamus_l | 22.5 ± 2.1 | 20.7 ± 1.1 | 0.0089 ± 0.0030 | 0.0101 ± 0.0024 | 0.093 ± 0.011 | 0.079 ± 0.007 | 27.3 ± 8.1 | 21.2 ± 4.2 |
| Cerebellum_l | 22.4 ± 2.0 | 20.6 ± 1.3 | 0.0061 ± 0.0027 | 0.0072 ± 0.0022 | 0.099 ± 0.019 | 0.086 ± 0.008 | 41.8 ± 14.5 | 30.5 ± 7.8 |

**Supplementary Table 10: Parameter estimates for subjects 1-5 (between-subject mean +/- s.d.) for [^13^N]ammonia data modelling, reversible 1TCM.**

| ROI | K1 (ml/min/100g) | | | k2 (1/min) | | | Vb | | | VT (ml/g) | | |
| --- | --- | --- | --- | --- | --- | --- | --- | --- | --- | --- | --- | --- |
|  | ICC | Var | AbsVar | ICC | Var | AbsVar | ICC | Var | AbsVar | ICC | Var | AbsVar |
| Grey Matter | -0.83 | -8 ± 14 | 10 ± 12 | 0.58 | 11 ± 38 | 28 ± 24 | -0.01 | -12 ± 16 | 15 ± 11 | 0.24 | -18 ± 33 | 24 ± 28 |
| White Matter | -0.89 | -7 ± 14 | 9 ± 11 | 0.57 | 12 ± 43 | 32 ± 27 | -0.18 | -12 ± 17 | 16 ± 12 | 0.19 | -19 ± 37 | 28 ± 29 |
| Hippocampus_l | -0.06 | -7 ± 14 | 10 ± 11 | 0.89 | 23 ± 27 | 25 ± 24 | -0.11 | -11 ± 23 | 17 ± 17 | 0.65 | -30 ± 24 | 32 ± 20 |
| OL_ling_G_l | -0.41 | -9 ± 15 | 10 ± 14 | 0.58 | 5 ± 30 | 24 ± 15 | 0.75 | -12 ± 12 | 13 ± 9 | 0.64 | -14 ± 21 | 20 ± 14 |
| G_cing_post_l | -0.68 | -7 ± 13 | 9 ± 11 | 0.58 | 19 ± 40 | 30 ± 31 | -0.39 | -10 ± 20 | 16 ± 14 | 0.14 | -26 ± 40 | 31 ± 35 |
| Putamen_l | -0.83 | -10 ± 15 | 12 ± 12 | 0.59 | 13 ± 44 | 34 ± 27 | 0.17 | -14 ± 19 | 17 ± 15 | 0.21 | -23 ± 39 | 31 ± 30 |
| Thalamus_l | -0.79 | -8 ± 15 | 10 ± 13 | 0.72 | 15 ± 23 | 24 ± 9 | -0.29 | -16 ± 17 | 18 ± 14 | 0.76 | -23 ± 14 | 23 ± 14 |
| Cerebellum_l | -0.69 | -8 ± 14 | 9 ± 13 | 0.61 | 19 ± 39 | 33 ± 25 | 0.5 | -13 ± 14 | 15 ± 11 | 0.23 | -27 ± 34 | 28 ± 32 |

**Supplementary Table 11: Parameter estimate repeatability for subjects 1-5 (between-subject mean +/- s.d.) for [^13^N]ammonia data modelling, reversible 1TCM.**

**Supplementary Figure 4.** **Graphical representation of best model choice for each of the 79 ROIs for each subject and scan.**

For each subplot regions are represented by rows, and four candidate models are shown as columns. The greyscale represents model choice, with most preferred shown in black (defined by the model fit with the lowest Akaike Information Criterion), within each region (for each subject and scan), with least favoured model show in white. Model K1 is the irreversible 1TCM, K1-2 is the reversible 1TCM, K1-3 is the irreversible 2TCM, and K1-4 is the reversible 2TCM. All candidate models included a blood fraction as a free parameter. Overall model K1-2 is the most favoured model (followed by K1-3, K1-4 and K1 in order). A number of regions for some subjects show alternative model preferences indicating the model choice is not robustly selected. It should be noted, however, that for most of the datasets that favour the K1-3 model, the k3 parameter is not estimable.

|  | K_1_ | k_2_ | k_3_ | V_b_ | Total TACs |
| --- | --- | --- | --- | --- | --- |
| n=5 | 779 | 452 | 54 | 779 | 790 |
| n=8 | 1253 | 926 | 216 | 1253 | 1264 |

**Supplementary Table 12:** **Summary of parameter estimability summary from fits to irreversible 2TCM**. Table shows number time activity curves (TACs) where specified parameter is estimated with a value significantly greater than zero (p<0.05, corresponding to proportional error of 39%). First row shows data from subjects 1-5 (5 subjects x 2 scans x 79 ROIs = 790 total). Second row shows data from all 8 subjects (1264 TACS). Nearly all TACs provide K1 estimates with less than 39% error. Only 54/790 were able to estimate k_3_ within this confidence.

|  | Irreversible 1TCM | Reversible 1TCM | Irreversible 2TCM | Reversible 2TCM | Total TACs |
| --- | --- | --- | --- | --- | --- |
| n=5 | 24 | 656 | 109 | 1 | 790 |
| n=8 | 24 | 936 | 303 | 1 | 1264 |

**Supplementary Table 13:** **Model preference summary from AIC.**

Table shows number time activity curves (TACs) preferring specified model using lowest AIC from 4 models. First row shows data from subjects 1-5 (5 subjects x 2 scans x 79 ROIs = 790 total). Second row shows data from all 8 subjects (1264 TACS). The majority of TACs fitted the reversible 1TCM best. While 109/790 showed preferential fits to irreversible 2TCM this was only matched across scan pairs in a majority of subjects (3/5) in one region: left hippocampus.

**Supplementary Figure 5:** **[^13^N]ammonia time activity curves and model fits for ROIs.**

Time activity curves for 8 ROIs are shown for test and retest datasets for all subject. Data points show TAC values, and lines show best fit lines to the irreversible 2TCM. Coloured points and lines show subjects 1-5 used in the main analysis (blue, green, red, magenta & cyan, for subjects 1-5 respectively). Grey symbols and lines (triangle, square, diamond for subjects 6-8 respectively) show subjects 6-8 (not included in main analysis). Regions shown: grey matter, white matter and the left (l) hippocampus, lingual gyrus (OL_ling_G), posterior cingulate gyrus (G_cing_post), putamen, thalamus, and cerebellum. TAC scale differences are apparent between test-retest pairs for some subjects (notably subjects 7 & 8: square & diamond symbols) though comparable difference between those blood input functions were also apparent indicating differences in administered dose.

**Supplementary Figure 6:** **[^15^O]water time activity curves and model fits for ROIs.**

Time activity curves for 8 ROIs are shown for test and retest datasets for all subject. Data points show TAC values, and lines show best fit lines to the CBF model. Coloured points and lines show subjects 1-5 used in the main analysis (blue, green, red, magenta & cyan, for subjects 1-5 respectively). Grey symbols and lines (triangle, square, diamond for subjects 6-8 respectively) show subjects 6-8. Regions shown: grey matter, white matter and the left (l) hippocampus, lingual gyrus (OL_ling_G), posterior cingulate gyrus (G_cing_post), putamen, thalamus, and cerebellum.


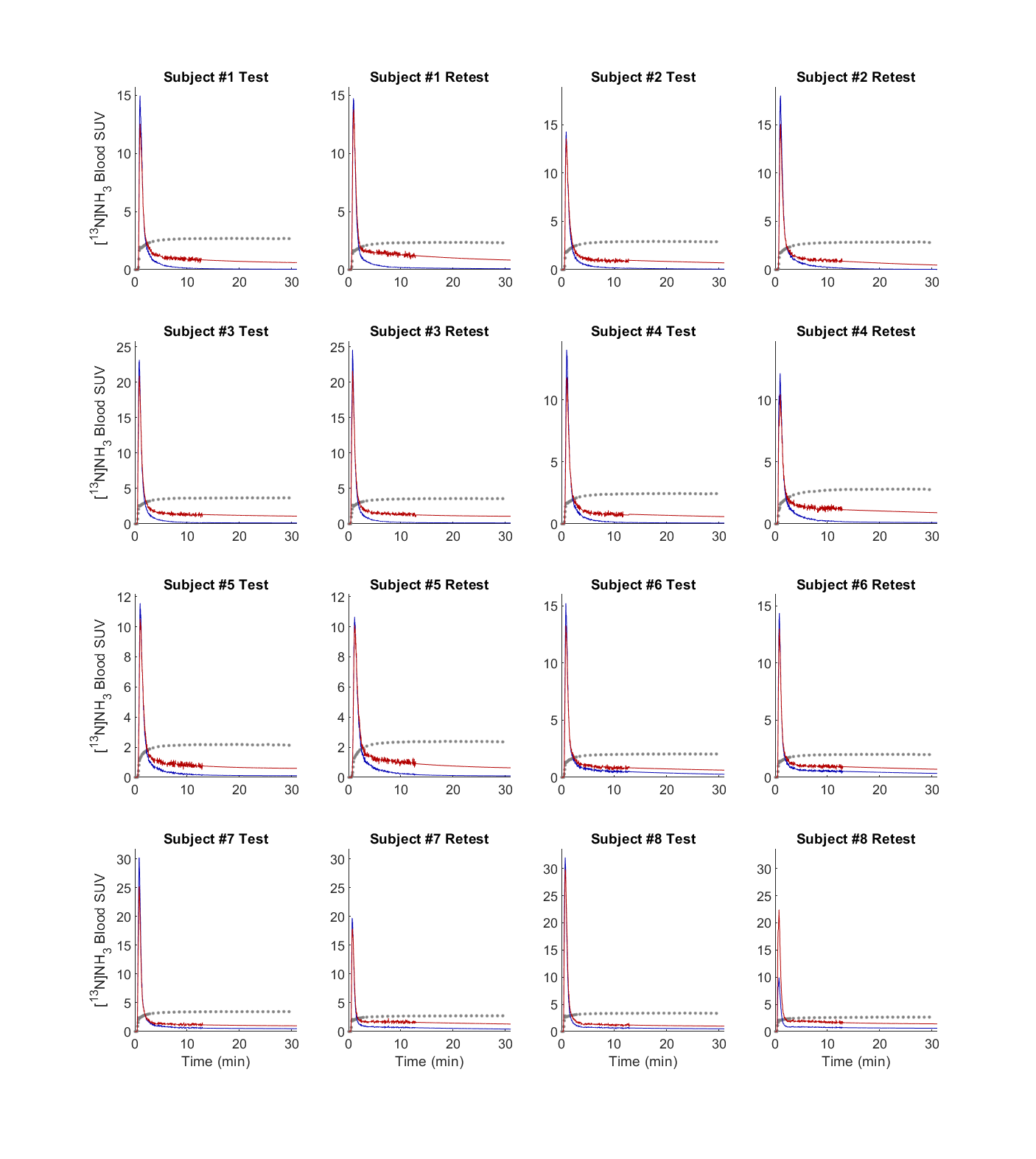


**Supplementary Figure 7:** **[^13^N]ammonia arterial blood and parent plasma input functions.**

Red lines shows whole arterial blood and blue parent plasma input functions for each subjects test and retest scans. Grey circles show grey matter TAC for comparison. The retest scans for subjects 7 & 8 show lower SUVs compared to the test scan. Since the relative change is also apparent in the tissue TAC, measured administered doses were almost identical and is also apparent in the paired [^15^O] scans it is possibly due to an undetected change in the venous cannula used for the tracer administration. The relatively low parent plasma concentration in subject 8’s retest scan is a consequence of the biexponential fit to the parent fraction data (see Figure 1, main manuscript).


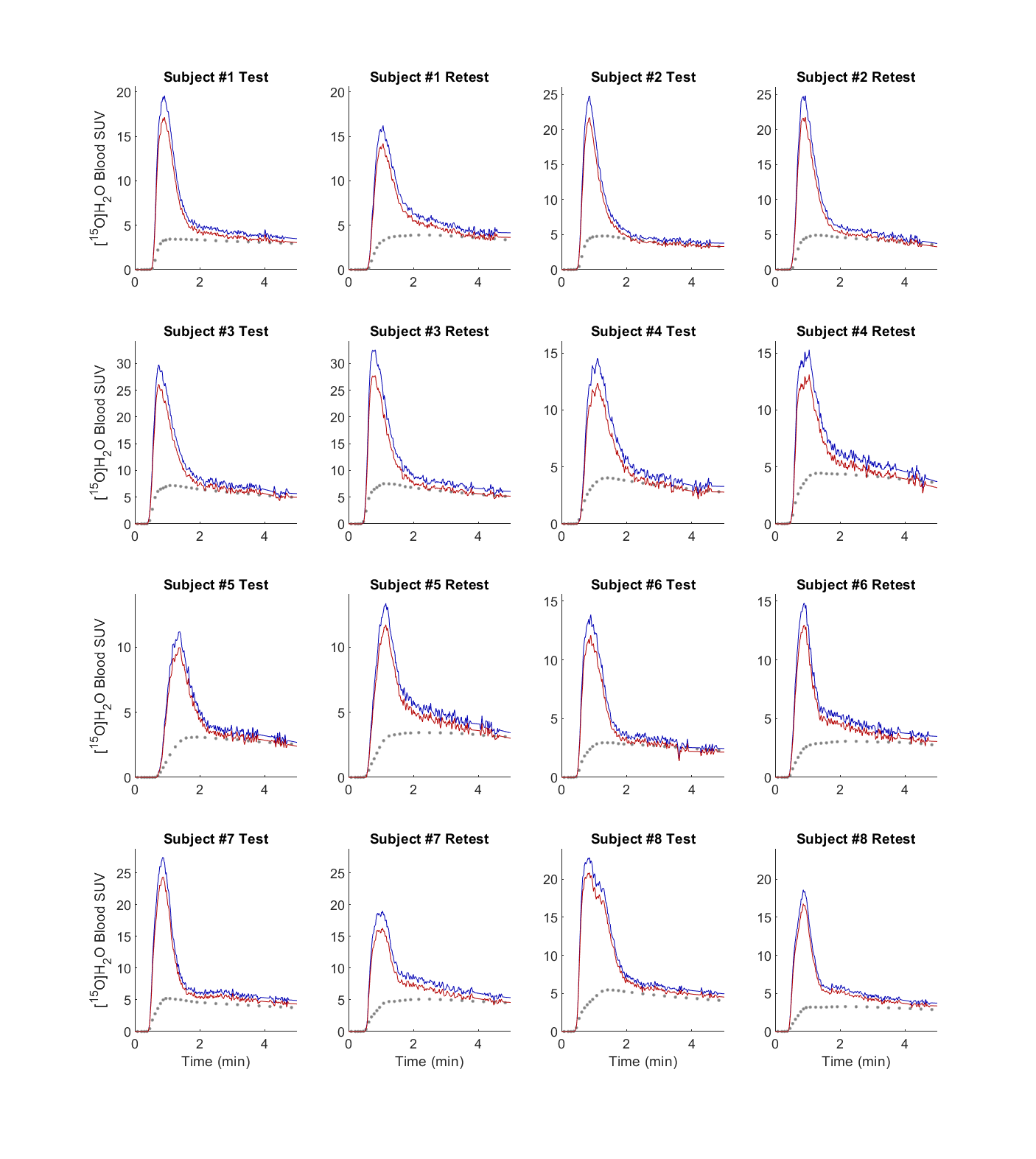


**Supplementary Figure 8:** **[^15^O]water arterial blood and plasma input functions.**

Red lines shows whole arterial blood and blue parent plasma input functions for each subjects test and retest scans. Grey circles show grey matter TAC for comparison. The retest scans for subjects 7 & 8 show lower SUVs compared to the test scan. Since the relative change is also apparent in the tissue TAC, measured administered doses were almost identical and is also apparent in the paired [^13^N] scans it is possibly due to an undetected change in the venous cannula used for the tracer administration.

**References**

1. Gluck MR, Thomas RG, Davis KL, Haroutunian V. Implications for altered glutamate and GABA metabolism in the dorsolateral prefrontal cortex of aged schizophrenic patients. Am J Psychiatry. 2002;159(7):1165-73. doi:10.1176/appi.ajp.159.7.1165.

2. Burbaeva G, Boksha IS, Turishcheva MS, Vorobyeva EA, Savushkina OK, Tereshkina EB. Glutamine synthetase and glutamate dehydrogenase in the prefrontal cortex of patients with schizophrenia. Prog Neuropsychopharmacol Biol Psychiatry. 2003;27(4):675-80. doi:10.1016/s0278-5846(03)00078-2.

3. Prabakaran S, Swatton JE, Ryan MM, Huffaker SJ, Huang JT, Griffin JL et al. Mitochondrial dysfunction in schizophrenia: evidence for compromised brain metabolism and oxidative stress. Mol Psychiatry. 2004;9(7):684-97, 43. doi:10.1038/sj.mp.4001511.

4. Bruneau EG, McCullumsmith RE, Haroutunian V, Davis KL, Meador-Woodruff JH. Increased expression of glutaminase and glutamine synthetase mRNA in the thalamus in schizophrenia. Schizophr Res. 2005;75(1):27-34. doi:10.1016/j.schres.2004.12.012.

5. Toro CT, Hallak JE, Dunham JS, Deakin JF. Glial fibrillary acidic protein and glutamine synthetase in subregions of prefrontal cortex in schizophrenia and mood disorder. Neurosci Lett. 2006;404(3):276-81. doi:10.1016/j.neulet.2006.05.067.

6. Burbaeva G, Boksha IS, Tereshkina EB, Savushkina OK, Starodubtseva LI, Turishcheva MS et al. Systemic neurochemical alterations in schizophrenic brain: glutamate metabolism in focus. Neurochem Res. 2007;32(9):1434-44. doi:10.1007/s11064-007-9328-7.

7. Steffek AE, McCullumsmith RE, Haroutunian V, Meador-Woodruff JH. Cortical expression of glial fibrillary acidic protein and glutamine synthetase is decreased in schizophrenia. Schizophr Res. 2008;103(1-3):71-82. doi:10.1016/j.schres.2008.04.032.

8. Martins-de-Souza D, Schmitt A, Roder R, Lebar M, Schneider-Axmann T, Falkai P et al. Sex-specific proteome differences in the anterior cingulate cortex of schizophrenia. J Psychiatr Res. 2010;44(14):989-91. doi:10.1016/j.jpsychires.2010.03.003.

9. Katsel P, Byne W, Roussos P, Tan W, Siever L, Haroutunian V. Astrocyte and glutamate markers in the superficial, deep, and white matter layers of the anterior cingulate gyrus in schizophrenia. Neuropsychopharmacology. 2011;36(6):1171-7. doi:10.1038/npp.2010.252.

10. Zhang L, Verwer RWH, Lucassen PJ, Huitinga I, Swaab DF. Prefrontal cortex alterations in glia gene expression in schizophrenia with and without suicide. J Psychiatr Res. 2020;121:31-8. doi:10.1016/j.jpsychires.2019.11.002.

11. Choudary PV, Molnar M, Evans SJ, Tomita H, Li JZ, Vawter MP et al. Altered cortical glutamatergic and GABAergic signal transmission with glial involvement in depression. Proc Natl Acad Sci U S A. 2005;102(43):15653-8. doi:10.1073/pnas.0507901102.

12. Kim S, Choi KH, Baykiz AF, Gershenfeld HK. Suicide candidate genes associated with bipolar disorder and schizophrenia: an exploratory gene expression profiling analysis of post-mortem prefrontal cortex. BMC Genomics. 2007;8:413. doi:10.1186/1471-2164-8-413.

13. Karolewicz B, Szebeni K, Gilmore T, Maciag D, Stockmeier CA, Ordway GA. Elevated levels of NR2A and PSD-95 in the lateral amygdala in depression. Int J Neuropsychopharmacol. 2009;12(2):143-53. doi:10.1017/S1461145708008985.

14. Sequeira A, Mamdani F, Ernst C, Vawter MP, Bunney WE, Lebel V et al. Global brain gene expression analysis links glutamatergic and GABAergic alterations to suicide and major depression. PLoS One. 2009;4(8):e6585. doi:10.1371/journal.pone.0006585.

15. Klempan TA, Sequeira A, Canetti L, Lalovic A, Ernst C, ffrench-Mullen J et al. Altered expression of genes involved in ATP biosynthesis and GABAergic neurotransmission in the ventral prefrontal cortex of suicides with and without major depression. Mol Psychiatry. 2009;14(2):175-89. doi:10.1038/sj.mp.4002110.

16. Kekesi KA, Juhasz G, Simor A, Gulyassy P, Szego EM, Hunyadi-Gulyas E et al. Altered functional protein networks in the prefrontal cortex and amygdala of victims of suicide. PLoS One. 2012;7(12):e50532. doi:10.1371/journal.pone.0050532.

17. Chandley MJ, Szebeni K, Szebeni A, Crawford J, Stockmeier CA, Turecki G et al. Gene expression deficits in pontine locus coeruleus astrocytes in men with major depressive disorder. J Psychiatry Neurosci. 2013;38(4):276-84. doi:10.1503/jpn.120110.

18. Zhao J, Verwer RW, van Wamelen DJ, Qi XR, Gao SF, Lucassen PJ et al. Prefrontal changes in the glutamate-glutamine cycle and neuronal/glial glutamate transporters in depression with and without suicide. J Psychiatr Res. 2016;82:8-15. doi:10.1016/j.jpsychires.2016.06.017.

19. Bernard R, Kerman IA, Thompson RC, Jones EG, Bunney WE, Barchas JD et al. Altered expression of glutamate signaling, growth factor, and glia genes in the locus coeruleus of patients with major depression. Mol Psychiatry. 2011;16(6):634-46. doi:10.1038/mp.2010.44.

20. Shimmura C, Suzuki K, Iwata Y, Tsuchiya KJ, Ohno K, Matsuzaki H et al. Enzymes in the glutamate-glutamine cycle in the anterior cingulate cortex in postmortem brain of subjects with autism. Mol Autism. 2013;4(1):6. doi:10.1186/2040-2392-4-6.

21. van der Hel WS, Notenboom RG, Bos IW, van Rijen PC, van Veelen CW, de Graan PN. Reduced glutamine synthetase in hippocampal areas with neuron loss in temporal lobe epilepsy. Neurology. 2005;64(2):326-33. doi:10.1212/01.WNL.0000149636.44660.99.

22. Eid T, Lee TS, Wang Y, Perez E, Drummond J, Lauritzen F et al. Gene expression of glutamate metabolizing enzymes in the hippocampal formation in human temporal lobe epilepsy. Epilepsia. 2013;54(2):228-38. doi:10.1111/epi.12008.

23. Matsuda-Matsumoto H, Iwazaki T, Kashem MA, Harper C, Matsumoto I. Differential protein expression profiles in the hippocampus of human alcoholics. Neurochem Int. 2007;51(6-7):370-6. doi:10.1016/j.neuint.2007.04.001.

24. Miguel-Hidalgo JJ, Waltzer R, Whittom AA, Austin MC, Rajkowska G, Stockmeier CA. Glial and glutamatergic markers in depression, alcoholism, and their comorbidity. J Affect Disord. 2010;127(1-3):230-40. doi:10.1016/j.jad.2010.06.003.

25. Carter CJ. Glutamine synthetase activity in Huntington's disease. Life Sci. 1982;31(11):1151-9. doi:10.1016/0024-3205(82)90090-x.

26. Burbaeva G, Boksha IS, Tereshkina EB, Savushkina OK, Starodubtseva LI, Turishcheva MS. Glutamate metabolizing enzymes in prefrontal cortex of Alzheimer's disease patients. Neurochem Res. 2005;30(11):1443-51. doi:10.1007/s11064-005-8654-x.

27. Lavoie J, Giguere JF, Layrargues GP, Butterworth RF. Activities of neuronal and astrocytic marker enzymes in autopsied brain tissue from patients with hepatic encephalopathy. Metab Brain Dis. 1987;2(4):283-90. doi:10.1007/bf00999698.

28. Cooper AJ. 13N as a tracer for studying glutamate metabolism. Neurochem Int. 2011;59(4):456-64. doi:10.1016/j.neuint.2010.11.011.

29. Keiding S, Sorensen M, Bender D, Munk OL, Ott P, Vilstrup H. Brain metabolism of 13N-ammonia during acute hepatic encephalopathy in cirrhosis measured by positron emission tomography. Hepatology. 2006;43(1):42-50. doi:10.1002/hep.21001.
